# A Nonlinear Elastic Model for Compressible Aluminum Alloys with Finite Element Implementation

**DOI:** 10.3390/ma14237351

**Published:** 2021-11-30

**Authors:** Aleksander Szwed, Marcin D. Gajewski

**Affiliations:** Faculty of Civil Engineering, Warsaw University of Technology, 00-637 Warsaw, Poland; m.gajewski@il.pw.edu.pl

**Keywords:** nonlinear elasticity, Green elasticity, deformation theory of plasticity, infinitesimal strain, specific energy, stress–strain relations, constitutive relationship, aluminum alloys, FEM simulations, buckling

## Abstract

In this paper, a three-dimensional model of nonlinear elastic material is proposed. The model is formulated in the framework of Green elasticity, which is based on the specific elastic energy potential. Equivalently, this model can be associated to the deformation theory of plasticity. The constitutive relationship, derived from the assumed specific energy, divides the material’s behavior into two stages: the first one starts with an initial almost linear stress–strain relation which, for higher strain, smoothly turns into the second stage of hardening. The proposed relation mimics the experimentally observed response of ductile metals, aluminum alloys in particular. In contrast to the classic deformation theory of plasticity or the plastic flow theory, the presented model can describe metal compressibility in both stages of behavior. The constitutive relationship is non-reversible expressing stress as a function of strain. Special attention is given to the calibration process, in which a one-dimensional analog of the three-dimensional model is used. Various options of calibration based on uniaxial stress test are extensively discussed. A finite element code is written and verified in order to validate the model. Solutions of selected problems, obtained via ABAQUS, confirm the correctness of the model and its usefulness in numerical simulations, especially for buckling.

## 1. Introduction

During an analysis of structural response under certain loading conditions, one of the issues to deal with is the definition of an appropriate constitutive relationship reflecting real behavior of the considered material. One-dimensional models based on uniaxial stress tests describe stress–strain relations, which can be used only in the cases of simple structural elements with a dominant uniaxial compressive or tensile stress state. Typical metal structures, as beams and columns, are usually composed of connected plate or shell elements. Under an acting load, some of those elements are in multiaxial stress states, which must be appropriately tied to multiaxial strain states. Therefore, there is a need for the definition of a suitable constitutive relation, more complex than the stress–strain relationship observed in a simple uniaxial test.

In case of metal structures, the currently used material models are formulated in the framework of either the deformation theory or the plastic flow theory. The deformation theory of plasticity (Hencky–Nadai), equivalent to the nonlinear infinitesimal elasticity theory of the Green type [1,2], can be used if the unloading process can be ignored and no significant redistribution of stresses due to permanent deformation is expected. On the other hand, the plastic flow theory (Lévy–Mises or Prandtl–Reuss) can be applied to calculations of structures under monotonic or cyclic loading [1,2]. A common issue of those theories for metals is that plastic strain does not allow any volumetric deformation, because it is governed by the second invariant of the stress deviator. However, there are plenty of models of elastic-plastic materials which include plastic volumetric deformation. They are suitable for frictional materials as soils, rocks, concrete and porous metals [1,2,3], but rarely for structural metals. The plastic flow theory leads to a path-dependent material response in which the actual strain depends on the history of previous deformation. The constitutive equations in the plastic flow theory are formulated in an incremental form in which the strain increment is determined by the current stress and its increment. According to this theory, the unloading takes place along a line parallel to the initial elastic path, preserving the initial stiffness of material. This is consistent with experimental behavior of most structural metals. Conversely, in the deformation theory of plasticity, the current state of stress is uniquely determined by the state of strain, and vice versa. The response is path-independent and the loading and unloading take place along the same nonlinear stress–strain path. Therefore, the deformation theory lacks the physical foundation compared to the plastic flow theory when permanent deformation occurs.

Despite this inconsistency, the deformation theory is used in many engineering problems involving inelastic (or nonlinear elastic) buckling of structures. The deformation theory seems to be more in agreement with experimental results than the plastic flow theory. This phenomenon is usually referred to as the “plastic buckling paradox” [4,5,6,7,8]. A more physically justified plastic flow theory leads generally to overestimated predictions of the critical load, whereas the application of the deformation theory of plasticity in buckling analysis delivers results more compatible with experimental data. This paradox has existed for many years leading to controversies, some of them are still to be resolved [4]. Numerous explanations of the issue were given in the literature but none of them seems to be completely satisfactory. For this reason, the deformation theory of plasticity is still recommended for practical engineering applications concerning the inelastic buckling of beams, columns, plates and shells [5,6,9]. Therefore, the analytical model presented in this paper refers to the deformation theory of plasticity, more precisely to the Green-type nonlinear elasticity.

Structural materials, such as aluminum alloys, high strength and stainless steel, exhibit a smooth nonlinear stress–strain response with no distinct yield limit. In majority of engineering applications, a piecewise stress–strain relationship consisting of a linear elastic, then a perfectly or hardening plastic response is used. The constitutive behavior of aluminum alloys in the uniaxial stress state can be adequately described by the Ramberg–Osgood law [10] and more accurately by its numerous piecewise enhancements, see in [11,12,13,14], among others. In the models, strain is given as an explicit, yet analytically non-invertible, function of stress [15]. This approach can be used in both the plastic flow theory and the deformation theory of plasticity. Such models can be used in hand calculations of simple structural elements [16], but solving more complex problems, especially handling finite element implementations, is problematic or impossible. Another group of constitutive models, suitable for the nonlinear elasticity theory (the deformation theory of plasticity), gives stress as an explicit function of strain. The constitutive relations are represented by smooth nonlinear curves, possibly piecewise, and, besides some simple cases, are non-invertible analytically [17,18,19,20]. When it comes to the development of three-dimensional models, they are based on various modifications of the distortional part of the linear constitutive relation to a nonlinear form, compare in [18,21]. In the plastic flow theory, the plastic strain rate is governed by the Huber–Mises yield function, leading to the incompressibility of plastic deformation, and by a continuous or a piecewise linear hardening law determined from experimental data. In the nonlinear elasticity (the deformation theory of plasticity), the distortional part of the linear constitutive relation is modified to a nonlinear form via a variable shear modulus. Therefore, this approach has the same limitations as the plastic flow theory. An attempt to resolve this issue is undertaken in [5], where variability of Poisson’s ratio is assumed, which, unfortunately, may violate thermodynamical restrictions. Furthermore, the deformation theory of plasticity formulated for the plane stress state and applied to the plate theory is used in [5,6,9], where interaction of buckling modes is investigated. Application of both theories in finite element programs can be found in many publications on stability of metallic structures, aluminum in particular, for example, in [22,23,24], among others. As a conclusion, there is an apparent lack of elastic models with fully nonlinear behavior for both the volumetric and distortional deformation with the inherently included variability of Young’s modulus and Poisson’s ratio, describing adequately compressible or nearly incompressible metals. Such models are common in the continuum mechanics (large strains) termed as hyperelastic or hyperplastic materials [21,25,26,27], but there is no direct transfer of those models to the infinitesimal elasticity theory, especially in the context of distinct requirements for convexity and material stability. 

In this paper, a nonlinear model for compressible and “plastically” incompressible metals is proposed. The definition of the model is based on the composition of two stages of material behavior. In the initial stage, the response is close to this of a linear elastic compressible material with certain stiffness, that is the well-known Hooke’s law. The second stage, denoted here as hardening, concerns advanced straining, physically plastic yield, which is modelled by a nonlinear constitutive relationship, which has an asymptotic stiffness different than initial one. The two relationships are smoothened to obtain a continuous transition between the mentioned limit responses. Then, the proposed model is calibrated with respect to experiments and implemented in the finite element method. 

After this introduction, the paper is organized as follows. In Section 2, the main part of the paper is given. We introduce an elastic strain energy potential, then, based on it, we derive a constitutive relationship and obtain a fourth-order tensor of tangent stiffness for incremental formulation. Moreover, we regard simplified models and analyze asymptotic properties of the model. All thermodynamic requirements applicable to nonlinear elastic materials are verified and limits for material constants are derived. Section 3 contains a calibration of the developed model. The calibration of material parameters is extensively discussed since the obtained constitutive relation is non-invertible and material stiffness varies with increasing strain. We show various approaches to the calibration, which is based on the uniaxial stress test. In Section 4, we describe the model’s implementation in a finite element code (ABAQUS) and show results of basic numerical tests in order to verify it. The validation in Section 5 includes more advanced examples of selected structural members. The most relevant research outcomes and conclusions are summarized in Section 6. 

## 2. Nonlinear Elastic Model for Isotropic Material

In this section, we propose and describe features a three-dimensional model of nonlinear elastic material, which can be regarded as a model in the framework of the deformation theory of plasticity. We start from a definition of an elastic specific energy potential, then derive a constitutive relationship and secant and tangent stiffnesses. The constitutive relationship is formulated in a format in which the stress tensor is a function of the strain tensor. We deliver graphical interpretations of the potential and the constitutive relation and derive limits for material constants to meet the thermodynamical requirements [1,2]. We reduce this model to the simplified case of a material which reflects “plastic” incompressibility (here termed as asymptotically incompressible) in the second stage of its behavior. This type of material is closely related to the Hencky–Nadai deformation theory of plasticity with modified distortional part of the constitutive relationship. Moreover, the Prandtl–Reuss plastic flow theory based on the Huber–Mises yield condition with an isotropic hardening can describe similar material response under monotonic loading programs, which do not result in significant redistribution of stresses due to plastic deformation.

### 2.1. Elastic Energy Function

We start the development of the constitutive relationship of an isotropic nonlinear elastic material with definition of the specific elastic energy function. An isotropic function of two strain tensor invariants $W\left(p,q\right)$ is assumed in the form:

(1) $$W=\frac{1}{2}\left(3Kp^{2}+2Gq^{2}\right)+\left(G_{0}-G\right)q_{0}^{2}\left(\frac{p^{2}}{p_{0}^{2}}+\frac{q^{2}}{q_{0}^{2}}\right)F\left[\frac{1}{2n},\frac{1}{n},1+\frac{1}{n},-{\left(\frac{p^{2}}{p_{0}^{2}}+\frac{q^{2}}{q_{0}^{2}}\right)}^{n}\right],\;F=1+\frac{ab}{c}z+\frac{a\left(1+a\right)b\left(1+b\right)}{2!c\left(1+c\right)}z^{2}+\frac{a\left(1+a\right)\left(2+a\right)b\left(1+b\right)\left(2+b\right)}{3!c\left(1+c\right)\left(2+c\right)}z^{3}+\ldots ,$$

where K, G, $G_{0}$, $q_{0}$, $p_{0}$, n are material constants and $F\left(a,b,c,z\right)$ is the hypergeometric function, which has an appropriate (hypergeometric) power series expansion [28]. We use the following notation for the strain tensor invariants:

(2) $$p=\frac{1}{\sqrt{3}}\mathrm{tr}\varepsilon ,\;q=\sqrt{\mathrm{tr}e^{2}}\geq 0,\;\mathrm{where}\;e=\varepsilon -pk$$

is the deviator of the infinitesimal strain tensor ε, and tr is the trace operation. The cubic (isotropic) tensor k is defined as $\sqrt{3}\;k=I$ with property $\left\|k\right\|=1$, while I is the unit second-order tensor. We use two invariants instead of all three invariants to keep simplicity of the model, the form of the considered strain energy is complex enough. 

In total, six independent constants are required to define this model, namely K, G, $G_{0}$, $q_{0}$, $p_{0}$, n. In the second stage, that is when the material is “plastically” incompressible, the number of material constants is reduced to five. The proposed elastic energy is a potential for stress [1,29] when the material constants involved in Equation (1) meet the following condition:

(3) $$2\left(G_{0}-G\right)q_{0}^{2}=3\left(K_{0}-K\right)p_{0}^{2},$$

where $K_{0}$ is an auxiliary material constant, that can be calculated from Equation (3) when other constants are known. 

In order to fulfil basic thermodynamic requirements [1,2,29,30], the specific elastic energy in Equation (1) has to be a non-negative $W\left(p,q\right)\geq 0$ and convex function [30]. Convexity conditions are:

(4) $$\frac{\partial^{\;2}W}{\partial p^{\;2}}\geq 0,\;\frac{\partial W}{\partial q}\geq 0,\;\frac{\partial^{\;2}W}{\partial q^{\;2}}\geq 0,\;\left(\frac{\partial^{\;2}W}{\partial p^{\;2}}\right)\left(\frac{\partial^{\;2}W}{\partial q^{\;2}}\right)-{\left(\frac{\partial^{\;2}W}{\partial p\partial q}\right)}^{2}\geq 0.$$

**Figure 1:**
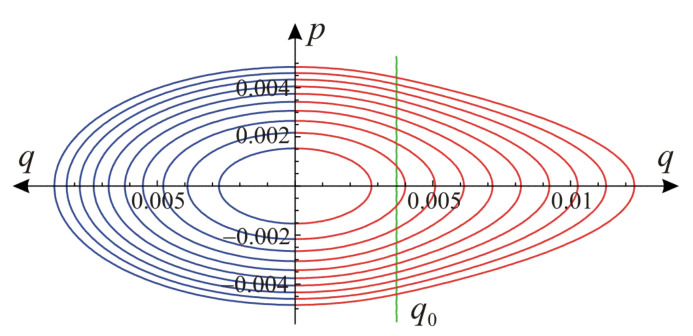
Contours of elastic strain energy for a linear material with $K_{0}=56,700\;\mathrm{MPa}$, $G_{0}=26,200\;\mathrm{MPa}$ (left part, blue contours), and for the nonlinear material with: $K_{0}=K=56,700\;\mathrm{MPa}$, $G_{0}=26,200\;\mathrm{MPa}$, G=100 MPa, $q_{0}=0.003688$, n=3 (right part, red contours).

**Figure 2:**
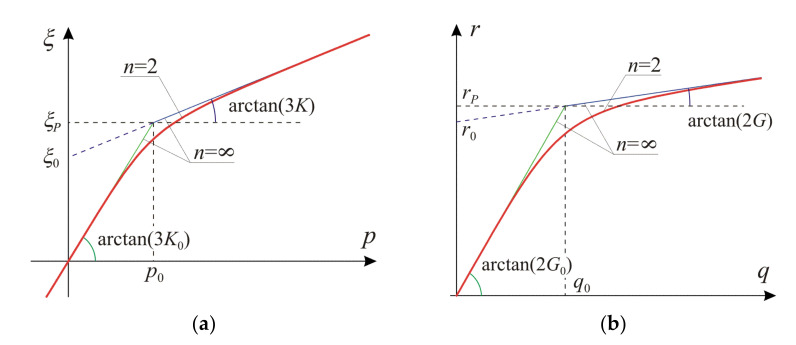
Relationships between stress and strain invariants and asymptotes for $K_{0}\;=\;4K$, $G_{0}\;=\;12G$: (**a**) p versus ξ for q=0; (**b**) q versus r for p=0. Interpretation of material parameters.

Note that the specific elastic energy in Equation (1) is supposed to be a strictly convex function, but here, we allow it to be only convex to leave doors open for investigation of limiting cases. To satisfy strict inequalities in Equation (4), the initial bulk and shear moduli defined at the strain origin $\left\|\varepsilon \right\|=0$ must be positive, that is $K_{0}>0$ and $G_{0}>0$, accordingly. The limit (or termed as asymptotic) bulk K and G shear moduli, which describe the material response for substantial strains ($\left\|\varepsilon \right\|=\sqrt{p^{2}+q^{2}}\to \infty$) called hardening, must be non-negative, K≥0 and G≥0. Moreover, the function in Equation (1) fulfils the natural state condition $W\left(0,0\right)=0$. Using Young’s moduli $E_{0}$ and E along with Poisson’s ratios $\nu_{0}$ and ν, the regarded bulk and shear moduli are expressed in the classic way as for linear elastic materials:

(5) $$K_{0}=\frac{E_{0}}{3\left(1-2\nu_{0}\right)},\;G_{0}=\frac{E_{0}}{2\left(1+\nu_{0}\right)}\;\mathrm{and}\;K=\frac{E}{3\left(1-2\nu \right)},\;G=\frac{E}{2\left(1+\nu \right)}$$

with appropriate limits applied to them: $E_{0}>0$, $-1<\nu_{0}<1/2$ and E>0; the limits for ν are specified below in Equation (7). Parameters $p_{0}\geq 0$ and $q_{0}\geq 0$ are the characteristic values of the strain invariants defined in Equation (2) and their interpretation is presented in Figure 1 and Figure 2. Power n (termed as a regularization parameter) is allowed to take any value higher than one half, n>1/2, but for metallic alloys, n≥1 is a suitable choice, and for aluminum alloys, n is typically between 2 and 6.

Besides the above-mentioned limits, the convexity conditions in Equation (4) are met when the initial material stiffness moduli are greater than the hardening moduli (so softening of the material is not allowed):

(6) $$K_{0}\geq K\;\mathrm{and}\;G_{0}\geq G.$$

Using Equation (5) and the limits set by Equation (6), the following restrictions on the hardening stage (asymptotic) Poisson’s ratio can be derived:

(7) $$\nu_{\min}=\left(1+\nu_{0}\right)\frac{E}{E_{0}}-1\leq \nu \leq \frac{1}{2}-\left(\frac{1}{2}-\nu_{0}\right)\frac{E}{E_{0}}=\nu_{\max}.$$

The initial values of Young’s modulus $E_{0}$ and Poisson’s ratio $\nu_{0}$ are well documented for elastic materials. Modulus E for the hardening part of the material response (physically with ongoing elastic-plastic deformation) can be determined from an experimental uniaxial test, while the value of Poisson’s ratio ν is postulated in the limits described in Equation (7). For aluminum alloys exhibiting plastic incompressibility, the values of ν can be assumed close to the upper limit given in Equation (7), compare in [5,31].

For certain values of the power n, the elastic strain energy can be expressed via elementary functions. For n=1, the formula from Equation (1) takes the following form:

(8) $$W\left(p,q\right)=\frac{1}{2}\left(3Kp^{2}+2Gq^{2}\right)+2\left(G_{0}-G\right)q_{0}^{2}\left[\sqrt{1+\frac{p^{2}}{p_{0}^{2}}+\frac{q^{2}}{q_{0}^{2}}}-1\right].$$

When $K\to K_{0}$ (or equivalently $\nu \to \nu_{\max}$) then $p_{0}\to \infty$ and the elastic energy defined in Equation (1) simplifies to:

(9) $$W\left(p,q\right)=\frac{1}{2}\left(3K_{0}p^{2}+2Gq^{2}\right)+\left(G_{0}-G\right)q^{2}F\left[\frac{1}{2n},\frac{1}{n},1+\frac{1}{n},-{\left(\frac{q^{2}}{q_{0}^{2}}\right)}^{n}\right].$$

which describes the linear elastic behavior for the isotropic part and hardening occurring only for the deviatoric part. This case of behavior is often assumed in the deformation theory of plasticity [5,9,21]. When K=0, G=0 and $q_{0}\to \infty$, Equations (1) and (8) reduce to the well-known formula for a linear material:

(10) $$W_{lin}\left(p,q\right)=\frac{1}{2}\left(3K_{0}p^{2}+2G_{0}q^{2}\right).$$

Comparison of the same contours of elastic energy for linear (Equation (10)) and nonlinear (Equation (1)) material models in the plane of invariants defined in Equation (2) is shown in Figure 1. Contours for the linear model (blue) are elliptic, while for the nonlinear material (red) they change shape with increasing strains from elliptic for the first phase of behavior to the shape of a rounded parallelogram for the second phase of material behavior. This shape is due to significant difference between values of the bulk and shear moduli for the hardening part of material’s response. The green line $q=q_{0}$ marks the border between two stages of material behavior described by the energy in Equation (9). 

### 2.2. Constitutive Relationship

The following constitutive relationship is derived according to the potential law of elasticity [1,2]:

(11) $$\sigma =\frac{\partial W}{\partial \varepsilon }=\frac{\partial W}{\partial p}\frac{\partial p}{\partial \varepsilon }+\frac{\partial W}{\partial q}\frac{\partial q}{\partial \varepsilon }.$$

Calculating the above derivatives of the function given in Equation (1) and of the strain invariants defined by Equation (2), we obtain the following result for the stress tensor:

(12) $$\sigma =3Kpk+2Gqd+\frac{1}{Q}\left[3\left(K_{0}-K\right)pk+2\left(G_{0}-G\right)qd\right],$$

with introduced additional notation for an increasing scalar-valued function of strain tensor invariants:

(13) $$Q\equiv Q\left(p,q\right)=\sqrt[{2n}]{1+{\left(\frac{p^{2}}{p_{0}^{2}}+\frac{q^{2}}{q_{0}^{2}}\right)}^{n}}=\sqrt[{2n}]{1+{\left(\frac{3\left(K_{0}-K\right)p^{2}}{2\left(G_{0}-G\right)q_{0}^{2}}+\frac{q^{2}}{q_{0}^{2}}\right)}^{n}}.$$

We use a normalized deviatoric tensor $\left\|d\right\|=1$, described by relation e= qd. Function Q defined by Equation (13) couples the isotropic and deviatoric parts of the constitutive relationship expressed by Equation (12). In case of stress tensor σ, we use invariants analogical as in Equation (2):

(14) $$\xi =\frac{1}{\sqrt{3}}\mathrm{tr}\sigma ,\;r=\sqrt{\mathrm{tr}s^{2}}\geq 0,\;\mathrm{where}\;s=\sigma -\xi k$$

is the stress tensor deviator. Based on Equation (12), the first stress tensor invariant can be expressed as:

(15) $$\xi =3Kp+3\left(K_{0}-K\right)\frac{p}{Q}=3K_{S}p,$$

and, in similar way, the second invariant of the stress deviator is of the form:

(16) $$r=2Gq+2\left(G_{0}-G\right)\frac{q}{Q}=2G_{S}q.$$

Introduced material functions $K_{S}\left(p,q\right)$ and $G_{S}\left(p,q\right)$ are the secant bulk and shear moduli, accordingly. Secant moduli are easily determined in experiments of various materials, so the above Equations (15) and (16) can be used for calibration of material parameters.

For various interpretations, it is convenient to introduce stress-type constants $\xi_{0}=3\left(K_{0}-K\right)p_{0}\geq 0$ and $r_{0}=2\left(G_{0}-G\right)q_{0}\geq 0$ connected to the stress tensor invariants (14) and rewrite Equation (3) as:

(17) $$2\left(G_{0}-G\right)q_{0}^{2}=3\left(K_{0}-K\right)p_{0}^{2}=\xi_{0}p_{0}=r_{0}q_{0}.$$

The relations between the stress tensor invariants and strain tensor invariants according to Equations (15) and (16) are shown in Figure 2. They are plotted for the selected ratios of the asymptotic and the initial moduli ($G/G_{0}$ and $K/K_{0}$). The considered nonlinear model is drawn for n=2 and for the asymptotic case n→∞, which determines the upper bound for the model predictions with respect to n. Power n can be viewed as a regularization parameter of a piecewise linear relation when n=∞. The asymptotes (n→∞) include the initial linear material envelope (green lines) and the hardening envelope (blue lines), compare with Equations (20) and (21). In addition, characteristic values of the stress and strain invariants, that is $p_{0}$, $q_{0}$ and $\xi_{0}$, $r_{0}$, are interpreted graphically. Moreover, parameters $\xi_{P}=K_{0}p_{0}$ and $r_{P}=G_{0}q_{0}$, defining location of the elbow points in the piecewise linear graphs, are explained.

Let us investigate the asymptotic properties of the proposed model, that is the case of n→∞. Function Q defined by Equation (13) is bound by the following:

(18) $$\underset{n\to \infty }{\lim}Q=1\;\mathrm{for}\;Q_{A}=\sqrt{\frac{p^{2}}{p_{0}^{2}}+\frac{q^{2}}{q_{0}^{2}}}\leq 1,$$

(19) $$\underset{n\to \infty }{\lim}Q=Q_{A}\;\mathrm{for}\;Q_{A}>1.$$

Condition $Q_{A}\left(p,q\right)\;=\;1$ defines an ellipse on the $\left(p,q\right)$ plane with the semi-axes $p_{0}$ and $q_{0}$. Then, the constitutive relation in Equation (12) is separated into two cases, that is the linear elastic envelope and the asymptotic relation:

(20) $$\sigma_{L}=3K_{0}pk+2G_{0}qd\;\mathrm{for}\;Q_{A}\leq 1\;\mathrm{and}$$

(21) $$\sigma_{A}=3Kpk+2Gqd+\frac{1}{Q_{A}}\left[3\left(K_{0}-K\right)pk+2\left(G_{0}-G\right)qd\right]\;\mathrm{for}\;Q_{A}>1.$$

Equation (20) describes the behavior of a linear material according to Equation (10) and establishes an envelope of the relation in Equation (12) for a strain fulfilling the condition $Q_{A}\leq 1$. For $Q_{A}=1$, the linear relation from Equation (20) switches to the asymptotic relation given in Equation (21). Interpretation of those relationships for the isotropic and deviatoric parts is shown on Figure 2. Results described by Equations (18) to (21) will be used effectively in the further model calibration process.

As a special case, we regard the simplified model described by Equation (9), which is defined by the proposed energy (Equation (1)) reduced for $K\to K_{0}$ and $p_{0}\to \infty$. As a result, the model includes five material constants. The constitutive relation in Equation (12) becomes:

(22) $$\sigma =3K_{0}pk+2Gqd+\frac{1}{Q_{I}}2\left(G_{0}-G\right)qd,\;\mathrm{and}\;Q_{I}=\sqrt[{2n}]{1+{\left(\frac{q^{2}}{q_{0}^{2}}\right)}^{n}}.$$

Then, Equations (15) and (16) for the above relation are:

(23) $$\xi =3K_{0}p\;\mathrm{and}\;r=2Gq+2\left(G_{0}-G\right)\frac{q}{Q_{I}}.$$

showing nonlinear material behavior only in the deviatoric part. For this case, the linear envelope relation in Equation (20) remains intact, while the asymptotic relation from Equation (21) changes as follows:

(24) $$\sigma_{A}=3K_{0}pk+2Gqd+\frac{1}{Q_{IA}}2\left(G_{0}-G\right)qd\;\mathrm{for}\;Q_{IA}=\sqrt{\frac{q^{2}}{q_{0}^{2}}}>1.$$

Equations (22)–(24) will be used in a simplified calibration procedure and for comparison with existing models.

### 2.3. Secant and Tangent Stiffnesses

In the finite element implementation, we use the rate (or incremental) constitutive relationships [1,2,21,25,26,27]. Let us now express Equation (12) in the following forms:

(25) $$\sigma =C_{S}.\varepsilon \;\mathrm{and}\;\dot{\sigma }=C_{T}.\dot{\varepsilon },$$

where $C_{S}$ is a fourth-order tensorial function of the secant stiffness, $C_{T}$ is a fourth-order tensorial function of the tangent stiffness of the material, while “.” stands for the full contraction operation and upper dot represents time derivative. Using tensor identities: $\left(k⊗k\right).\varepsilon =pk$ and $\left(1-k⊗k\right).\varepsilon =qd$, the secant stiffness is defined as:

(26) $$C_{S}=3K\;k⊗k+2G\left(1-k⊗k\right)+\frac{1}{Q}\left[3\left(K_{0}-K\right)k⊗k+2\left(G_{0}-G\right)\left(1-k⊗k\right)\right].$$

⊗ denotes the tensor product, 1 is the fourth-order unit tensor, that in any orthonormal basis $\left\{b_{i}\right\}$ has the components: $1_{ijkl}=\left(\delta_{ik}\delta_{jl}+\delta_{il}\delta_{jk}\right)/2$ for i,j,k,l=1,2,3. The tangent stiffness tensor is defined as a derivative of function in Equation (12) with respect to the strain tensor:

(27) $$\begin{array}{cc} C_{T}=\frac{\partial \sigma }{\partial \varepsilon }=C_{S} & -\frac{1}{Q^{2n+1}}{\left(\frac{p^{2}}{p_{0}^{2}}+\frac{q^{2}}{q_{0}^{2}}\right)}^{n-1}\left[3\left(K_{0}-K\right)\frac{p^{2}}{p_{0}^{2}}k⊗k\right.\\ & \left.+3\left(K_{0}-K\right)\frac{pq}{q_{0}^{2}}\left(d⊗k+k⊗d\right)+2\left(G_{0}-G\right)\frac{q^{2}}{q_{0}^{2}}d⊗d\right], \end{array}$$

where Equation (3) was used during derivations. Both tensorial functions expressed by Equation (26) and Equation (27) have all minor and mayor symmetries $C_{ijkl}=C_{jikl}=C_{ijlk}=C_{klij}$ for hyperelastic (Green type) materials [1,2,21,29]. The bulk and shear tangent stiffness functions are then written as:

(28) $$3K_{T}=3K_{S}-3\left(K_{0}-K\right)\frac{p^{2}}{p_{0}^{2}Q^{2n+1}}{\left(\frac{p^{2}}{p_{0}^{2}}+\frac{q^{2}}{q_{0}^{2}}\right)}^{n-1},$$

(29) $$2G_{T}=2G_{S}-2\left(G_{0}-G\right)\frac{q^{2}}{q_{0}^{2}Q^{2n+1}}{\left(\frac{p^{2}}{p_{0}^{2}}+\frac{q^{2}}{q_{0}^{2}}\right)}^{n-1}.$$

It is evident that the bulk or shear tangent stiffness is lower than the secant counterpart for a given strain level. When tangent stiffnesses are non-negative, the elastic energy described by Equation (1) is a convex function [29].

When $\left\|\varepsilon \right\|\to 0$, the functions of the secant $C_{S}\left(\varepsilon \right)$ and tangent $C_{T}\left(\varepsilon \right)$ stiffnesses take the same initial values:

(30) $$C_{S0}=C_{T0}=3K_{0}\;k⊗k+2G_{0}\left(1-k⊗k\right).$$

In case of $\left\|\varepsilon \right\|\to \infty$, both functions approach the following values of asymptotic stiffnesses:

(31) $$C_{SA}=C_{TA}=3K\;k⊗k+2G\left(1-k⊗k\right).$$

Expressions in Equation (30) and Equation (31) are identical to the stiffnesses of appropriate linear materials.

Notice that the scalar valued function $Q\left(p,q\right)$ defined by Equation (13) provides a smooth and continuous transition of the actual material stiffness from the initial value to the asymptotic one when 1/2<n<∞. This property allows to define independently material constants $K_{0}\;$ and $G_{0}$ for the initial stage of the material and constants K  and G for the advanced strain values (within the second stage), close to the material’s failure.

For the special case of $K\to K_{0}$ and $p_{0}\to \infty$, the stiffness tensors are:

(32) $$C_{S}=3K_{0}k⊗k+2G\left(1-k⊗k\right)+\frac{1}{Q_{I}}2\left(G_{0}-G\right)\left(1-k⊗k\right),$$

(33) $$C_{T}=C_{S}-\frac{1}{Q_{I}^{2n+1}}{\left(\frac{q^{2}}{q_{0}^{2}}\right)}^{n}2\left(G_{0}-G\right)d⊗d.$$

Then, the bulk and shear tangent stiffness functions are expressed as:

(34) $$3K_{T}=3K_{S}=3K_{0},\;2G_{T}=2G_{S}-2\left(G_{0}-G\right)\frac{1}{Q_{I}^{2n+1}}{\left(\frac{q^{2}}{q_{0}^{2}}\right)}^{n}.$$

## 3. Calibration of Material Parameters

The most convenient way to calibrate the developed model with respect to experimental tests is to use the pure shear stress and the equal triaxial tension or compression. Unfortunately, triaxial tests are almost impossible to perform, considering their complexity and expensiveness. The shear stress tests with ξ=0 can be carried out, but still are not a common practice in engineering applications. Technically, the uniaxial tension or the uniaxial compression tests are available for many materials, including aluminum alloys [10,11,12]. Typically, the uniaxial tests provide only a relation between the direct strain and the applied stress, with no relation for the transverse strain, whose availability can significantly improve the quality of calibration. The constitutive relationships for three-dimensional nonlinear models are usually quite complex and non-invertible, thus difficult to calibrate on the basis of sole uniaxial tests. Note, that the constitutive relationship in which the stress tensor is a function of the strain tensor is convenient for the finite element implementation, but is troublesome when the calibration is based on stress tests. In this section, first, we propose a calibration procedure, applicable to “plastically” incompressible material, which leads to an acceptable accuracy. Then, we describe a more general approach to calibration for fully compressible material and discuss the obtained results. We will use a one-dimensional model analog of the spatial one to approximate experimental results for the uniaxial tension or compression stress states.

### 3.1. One-Dimensional Analog of the Proposed Nonlinear Elastic Material Model

The elastic strain energy for the one-dimensional nonlinear elastic model is:

(35) $$W\left(\varepsilon \right)=\frac{1}{2}E\varepsilon^{2}+\left(E_{0}-E\right)\varepsilon^{2}F\left[\frac{1}{2n},\frac{1}{n},1+\frac{1}{n},-{\left(\frac{\varepsilon^{2}}{\varepsilon_{0}^{2}}\right)}^{n}\right],$$

where $F\left(a,b,c,z\right)$ is the hypergeometric function [28], see also Equation (1). The regarded specific energy function is non-negative, $W\left(0\right)=0$, and convex when $E_{0}>0$, $\varepsilon_{0}\geq 0$, $E_{0}\geq E$ and n>1/2. Function in Equation (35) depends on four material parameters: $E_{0}$, E, $\varepsilon_{0}$ and n which can be determined from the uniaxial tests. We define two useful stress parameters $\sigma_{0}\geq 0$ and $\sigma_{P}\geq 0$ by the following relations: $\sigma_{0}=\left(E_{0}-E\right)\varepsilon_{0}$ and $\sigma_{P}=E_{0}\varepsilon_{0}$. Differentiation of Equation (35) leads to the one-dimensional constitutive relationship:

(36) $$\sigma =E\varepsilon +\frac{1}{Q_{1}}\left(E_{0}-E\right)\varepsilon ,\;\mathrm{where}\;Q_{1}\left(\varepsilon \right)=\sqrt[{2n}]{1+{\left(\frac{\varepsilon^{2}}{\varepsilon_{0}^{2}}\right)}^{n}}.$$

When n→∞, the above relationship can be written in intervals as a piecewise linear relation:

(37) $$\sigma_{L}=E_{0}\varepsilon \;\mathrm{for}\;Q_{1A}\leq 1\;\mathrm{and}$$

(38) $$\sigma_{A}=E\varepsilon +\frac{1}{Q_{1A}}\left(E_{0}-E\right)\varepsilon \;\mathrm{for}\;Q_{1A}>1\;\mathrm{where}\;Q_{1A}=\sqrt{\frac{\varepsilon^{2}}{\varepsilon_{0}^{2}}}.$$

Equation (38) defines two skew asymptotes, one for tension and another for compression:

(39) $$\sigma_{AT}=E\varepsilon +\sigma_{0}\;\mathrm{and}\;\sigma_{AC}=E\varepsilon -\sigma_{0}.$$

**Figure 3:**
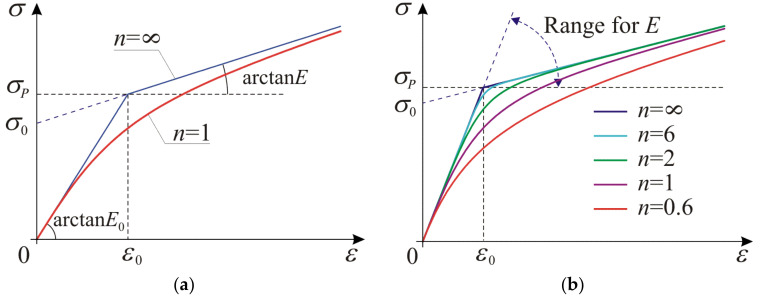
(**a**) Stress–strain curves according to the one-dimensional model of elastic material for n=1 and n→∞ (piecewise linear). Graphical interpretation of material parameters: $E_{0}$, E, $\varepsilon_{0}$, $\sigma_{0}$ and $\sigma_{P}$. (**b**) Stress–strain curves for several values of n with possible range for the hardening modulus E.

Graphs of the constitutive relation described by Equation (36) for n=1 with asymptotes according to Equations (37) and (38) for tension are shown in Figure 3. n can be interpreted as a parameter governing the smooth regularization of the piecewise linear relationship. All the material parameters introduced in the one-dimensional model are explained in Figure 3a. Range for the hardening modulus $0<E\leq E_{0}$ and stress–strain curves for several values of n>1/2 with fixed other parameters are presented in Figure 3b. The figure delivers a general overview on the flexibility of the proposed model in description of possible material’s response.

The secant and tangent stiffness functions are expressed by the formulae:

(40) $$E_{S}=\frac{\sigma }{\varepsilon }=E+\frac{1}{Q_{1}}\left(E_{0}-E\right)\;\mathrm{and}\;E_{T}=\frac{d\sigma }{d\varepsilon }=E+\frac{1}{Q_{1}^{2n+1}}\left(E_{0}-E\right).$$

Strict convexity of energy defined by Equation (35) with respect to strain is attained if $E_{T}\left(\varepsilon \right)>0$ for arbitrary ε. When ε→0, we obtain the initial stiffnesses $E_{S0}=E_{T0}=E_{0}$, and for ε→±∞, we get the asymptotic stiffness values $E_{SA}=E_{TA}=E$. Via the function $Q_{1}\left(\varepsilon \right)$, we can control the curvature of the elbow in the vicinity of $\left(\varepsilon_{0},\sigma_{P}\right)$ point shown in Figure 3. For low-hardening aluminum alloys, we observe a significant difference between the initial and the asymptotic stiffnesses, typically $E_{0}≅300E$, while the regularization parameter n takes values from 2 to 6 depending on the alloy type. During the presented calibration of the model, first, we estimate the initial modulus $E_{0}$ and the hardening modulus E, then calculate characteristic stress $\sigma_{0}$ (or strain $\varepsilon_{0}$) and, finally, we obtain the power n parameter numerically. In the literature, we can find various one-dimensional models which are calibrated with available experimental data, compare in [7,8,9,10,11,12,13]. The presented one-dimensional model can be calibrated with the same uniaxial tests. In case of one-dimensional model optimization techniques, such as the least squares method, can be used to enhance agreement with experimental data. However, those results cannot be directly transferred to calibration of the three-dimensional model against the uniaxial test.

### 3.2. Uniaxial Stress State

Typically, the calibration of model parameters is carried out based on the uniaxial tension (or compression) test, which is described by the following matrix representation of the stress and strain tensors in an orthonormal basis:

(41) $$\left[\sigma \right]=\left[\begin{array}{ccc} \sigma & 0 & 0\\ 0 & 0 & 0\\ 0 & 0 & 0 \end{array}\right]\;\mathrm{and}\;\left[\varepsilon \right]=\left[\begin{array}{ccc} \varepsilon & 0 & 0\\ 0 & \varepsilon_{T} & 0\\ 0 & 0 & \varepsilon_{T} \end{array}\right],$$

where σ and ε represent the normal stress and the direct strain in loading direction, and $\varepsilon_{T}$ are the transverse strains of an isotropic material. Based on the tension test σ≥0 and representations in Equation (41), the strain invariants from Equation (2) are:

(42) $$p=\frac{\varepsilon +2\varepsilon_{T}}{\sqrt{3}}\;\mathrm{and}\;q=\sqrt{\frac{2}{3}}\left(\varepsilon -\varepsilon_{T}\right).$$

Two independent scalar relationships resulting from Equation (12) take the form:

(43) $$\sigma =K\left(\varepsilon +2\varepsilon_{T}\right)+\frac{4}{3}G\left(\varepsilon -\varepsilon_{T}\right)+\frac{1}{Q_{UX}}\left[\left(K_{0}-K\right)\left(\varepsilon +2\varepsilon_{T}\right)+\frac{4}{3}\left(G_{0}-G\right)\left(\varepsilon -\varepsilon_{T}\right)\right],$$

(44) $$0=K\left(\varepsilon +2\varepsilon_{T}\right)-\frac{2}{3}G\left(\varepsilon -\varepsilon_{T}\right)+\frac{1}{Q_{UX}}\left[\left(K_{0}-K\right)\left(\varepsilon +2\varepsilon_{T}\right)-\frac{2}{3}\left(G_{0}-G\right)\left(\varepsilon -\varepsilon_{T}\right)\right],$$

where

(45) $$Q_{UX}=\sqrt[{2n}]{1+{\left[\frac{\left(K_{0}-K\right){\left(\varepsilon +2\varepsilon_{T}\right)}^{2}}{2\left(G_{0}-G\right)q_{0}^{2}}+\frac{2{\left(\varepsilon -\varepsilon_{T}\right)}^{2}}{3q_{0}^{2}}\right]}^{n}}.$$

The bulk and shear moduli are defined by Equation (5) via Young’s moduli and Poisson’s ratios. Since Equation (44) is highly nonlinear, the transverse strain $\varepsilon_{T}$ cannot be calculated directly from it. In such circumstances, it is not possible to substitute the result from Equation (44) into Equation (43) and obtain $\sigma \left(\varepsilon \right)$. Thus, there is no direct connection between the one-dimensional model relation and the uniaxial test that resulted from the three-dimensional one and typical curve-fitting techniques cannot be applied to calibrate the model. Instead, in the proposed simplified procedure, we will use the limiting relations from Equations (20) and (21) to obtain the material parameters.

In case of the linear envelope described by Equation (20), we get the well-known results:

(46) $$\sigma_{L}=K_{0}\left(\varepsilon +2\varepsilon_{T}\right)+\frac{4}{3}G_{0}\left(\varepsilon -\varepsilon_{T}\right)\;\mathrm{and}\;0=K_{0}\left(\varepsilon +2\varepsilon_{T}\right)-\frac{2}{3}G_{0}\left(\varepsilon -\varepsilon_{T}\right).$$

Application of Equation (5) in the solution of Equation (46) yields:

(47) $$\sigma_{L}=\frac{9K_{0}G_{0}}{G_{0}+3K_{0}}\varepsilon =E_{0}\varepsilon \;\mathrm{and}\;\varepsilon_{T}=\frac{2G_{0}-3K_{0}}{2\left(G_{0}+3K_{0}\right)}\varepsilon =-\nu_{0}\varepsilon .$$

### 3.3. Calibration Procedure for Simplified Model

First, we regard a special case where $K=K_{0}$ (or equivalently $\nu =\nu_{\max}$) and $G\neq G_{0}$ that results in $p_{0}\to \infty$. In other words, we keep a linear material behavior for the isotropic part, while the reduction of stiffness occurs for the distortional part of the constitutive relationship in Equation (22). We analyze this case separately because of its close relations to the Hencky–Nadai deformation theory of plasticity with modified deviatoric part of the constitutive relationship. We use results obtained here to compare the proposed model predictions with the Prandtl–Reuss plastic flow theory based on the Huber–Mises yield condition (plastic incompressibility) with an isotropic hardening [1,2,21].

For the uniaxial test according to Equation (41), the asymptotic relation defined in Equation (24) becomes:

(48) $$\sigma_{A}=K_{0}\left(\varepsilon +2\varepsilon_{T}\right)+\frac{4}{3}G\left(\varepsilon -\varepsilon_{T}\right)+\sqrt{\frac{8}{3}}\left(G_{0}-G\right)q_{0}\frac{\left(\varepsilon -\varepsilon_{T}\right)}{\sqrt{{\left(\varepsilon -\varepsilon_{T}\right)}^{2}}},$$

(49) $$0=K_{0}\left(\varepsilon +2\varepsilon_{T}\right)-\frac{2}{3}G\left(\varepsilon -\varepsilon_{T}\right)-\sqrt{\frac{2}{3}}\left(G_{0}-G\right)q_{0}\frac{\left(\varepsilon -\varepsilon_{T}\right)}{\sqrt{{\left(\varepsilon -\varepsilon_{T}\right)}^{2}}}.$$

Equation (49) can be solved analytically for the tension or compression test separately. In case of tension, we express the transverse strain as:

(50) $$\varepsilon_{T}=\sqrt{\frac{3}{2}}\frac{G_{0}-G}{G+3K_{0}}q_{0}+\frac{2G-3K_{0}}{2\left(G+3K_{0}\right)}\varepsilon$$

and, then, Equation (48) takes the form:

(51) $$\sigma_{A}=\sqrt{6}\;q_{0}\frac{3K_{0}\left(G_{0}-G\right)}{G+3K_{0}}+\frac{9GK_{0}}{G+3K_{0}}\varepsilon .$$

Comparison of the one-dimensional model asymptote expressed by Equation (39) for tension with Equation (51) yields the following calibration formulae for G and $q_{0}$:

(52) $$G=\frac{3EK_{0}}{9K_{0}-E}=\frac{E_{0}E}{3E_{0}-\left(1-2\nu_{0}\right)E}\;\mathrm{and}\;q_{0}=\frac{G+3K_{0}}{3K_{0}\left(G_{0}-G\right)}\frac{\sigma_{0}}{\sqrt{6}}.$$

Based on Equations (47) and (51), we can correlate $q_{0}$ and $\varepsilon_{0}$ solving $\sigma_{L}\left(\varepsilon_{0}\right)=\sigma_{A}\left(\varepsilon_{0}\right)$, which results in the following expression:

(53) $$q_{0}=\sqrt{\frac{3}{2}}\frac{3K_{0}\varepsilon_{0}}{G_{0}+3K_{0}}=\sqrt{\frac{2}{3}}\left(1+\nu_{0}\right)\varepsilon_{0}.$$

The first result in Equation (52) and the above outcome (Equation (53)) are the sought final calibration formulae. Having determined $E_{0}$ and E, we obtain $\sigma_{0}$ (or $\varepsilon_{0}$) in the one-dimensional model. Assuming $\nu_{0}$, we obtain $K_{0}$ and $G_{0}$ using Equation (5), then applying Equation (52) and Equation (53), we can calculate the G and $q_{0}$ parameters of the three-dimensional model.

Using Equation (50), we can define the asymptotic Poisson ratio function for the regarded simplified model:

(54) $$\nu_{AK}\left(\varepsilon \right)=-\frac{\varepsilon_{T}}{\varepsilon }=\frac{3K_{0}-2G}{2\left(G+3K_{0}\right)}-\sqrt{\frac{3}{2}}\frac{G_{0}-G}{G+3K_{0}}\frac{q_{0}}{\varepsilon }=\nu -\left(\frac{1}{2}-\nu_{0}\right)\left(1-\frac{E}{E_{0}}\right)\frac{\varepsilon_{0}}{\varepsilon },$$

which can be written in the alternative forms:

(55) $$\nu_{AK}\left(\varepsilon \right)=\frac{1}{2}-\left(\frac{1}{2}-\nu_{0}\right)\left[\frac{E}{E_{0}}+\left(1-\frac{E}{E_{0}}\right)\frac{\varepsilon_{0}}{\varepsilon }\right]=\nu +\left(\frac{1}{2}-\nu \right)\left(1-\frac{E_{0}}{E}\right)\frac{\varepsilon_{0}}{\varepsilon }.$$

Function $\nu_{AK}\left(\varepsilon \right)$ defines an envelope (the lower limit) for the actual Poisson’s ratio in the proposed model, compare with the results in [5,31]. Note that full incompressibility of the material is attained for ε→∞.

Let us summarize the sequence of calculations in the procedure of material parameters calibration for the simplified model. In the procedure, we assume the initial Poisson ratio $\nu_{0}$ and estimate (calculate) the initial $E_{0}$ and hardening E moduli from uniaxial experimental test data. The initial modulus $E_{0}$ should be determined as a trend line at the initial part of an experimental stress–strain curve. The hardening modulus E is estimated from the pre-peak part (hardening stage) of an experimental test. Then, we calculate $K_{0}$, $G_{0}$, G, $\sigma_{0}$, $\varepsilon_{0}$ and $q_{0}$ using the derived calibration formulae. The last parameter n, we determine from Equations (43) and (44) with $K=K_{0}$ based on one experimental point located in the vicinity of the elbow of an experimental stress–strain curve.

Constitutive relationships for the uniaxial stress state (Equations (43) and (44)) can be expressed in a parametric way using the generalized (for the model) Poisson ratio $\bar{\nu }$ defined by the equality $\varepsilon_{T}=-\bar{\nu }\;\varepsilon$. In the range of $\bar{\nu }$ prescribed by Equation (7), the relation between $\varepsilon \left(\bar{\nu }\right)$ and $\sigma \left(\bar{\nu }\right)$ is of the form:

(56) $$\sigma \left(\bar{\nu }\right)=3K_{0}\left(1-2\bar{\nu }\right)\varepsilon \left(\bar{\nu }\right),\;\varepsilon \left(\bar{\nu }\right)=\sqrt{\frac{3}{2}}\frac{q_{0}}{1+\bar{\nu }}\sqrt[{2n}]{{\left[\frac{2\left(G_{0}-G\right)\left(1+\bar{\nu }\right)}{3K_{0}\left(1-2\bar{\nu }\right)-2G\left(1+\bar{\nu }\right)}\right]}^{2n}-1}.$$

As an example, we use our own experimental test data for aluminum AW6063 T66. For the data, the conventional proportionality limit is estimated as $\sigma_{H}=145.1\;\mathrm{MPa}$ for strain $\varepsilon_{H}=0.002131$ and, then, the initial elasticity modulus is calculated as $E_{0}=\sigma_{H}/\varepsilon_{H}≅68,100\;\mathrm{MPa}$. For the estimation of hardening modulus, we select two points: in the initial hardening zone $\left(\varepsilon_{1},\sigma_{1}\right)=\left(0.01,238.6\;\mathrm{MPa}\right)$ and the ultimate stress $\left(\varepsilon_{U},\sigma_{U}\right)=\left(0.06064,253.9\;\mathrm{MPa}\right)$. Based on those values, we calculate parameters included in the one-dimensional model (Equation (36)):

(57) $$E=\frac{\sigma_{U}-\sigma_{1}}{\varepsilon_{U}-\varepsilon_{1}}≅300\;\mathrm{MPa},\;\sigma_{0}=\sigma_{1}-E\varepsilon_{1}=235.6\;\mathrm{MPa}\;\mathrm{and}$$

(58) $$\varepsilon_{0}=\frac{\sigma_{0}}{E_{0}-E}=0.003475,\;\sigma_{P}=E_{0}\varepsilon_{0}=236.6\;\mathrm{MPa}.$$

Assuming a value of the initial Poisson’s ratio of $\nu_{0}=0.3$, we obtain parameters of the three-dimensional model using Equations (5), (52) and (53):

(59) $$K_{0}=56,700\;\mathrm{MPa},\;G_{0}=26,200\;\mathrm{MPa},\;G=100\;\mathrm{MPa},\;q_{0}=0.003688.$$

Having G and $K=K_{0}$, we calculate the value of the asymptotic Poisson ratio $\nu =\nu_{\max}=0.499$ using the inverse form of the last relation in Equation (5), which is within the allowed limits according to Equation (7): −0.994≤ν≤0.499. The regularization parameter n is calculated using an additional calibration point located in the vicinity of elbow on the experimental stress–strain curve $\left(\varepsilon_{n}=\varepsilon_{0},\sigma_{n}\right)=\left(0.003475,214.5\;\mathrm{MPa}\right)$. Numerical solution to the system of Equations (43) and (44) via Wolfram Mathematica gives the following results: $\varepsilon_{T}=-0.001107$ and n=3.28≅3. Finite element simulations of test problems for those values of parameters are presented in the next sections. 

Since the one-dimensional model cannot be exactly transferred to the uniaxial state from the spatial one, the proposed calibration process is sensitive to the selection of calibration points. Results of calibration for several locations of the point in the hardening zone of the experimental curve $\left(\varepsilon_{1},\sigma_{1}\right)$ for determination of parameters $E_{0}$, E and $\sigma_{0}$ (or $\varepsilon_{0}$) are given in Table 1. Moreover, we can observe that the selection of $\left(\varepsilon_{n},\sigma_{n}\right)$ has a strong influence on the regularization parameter n as well. To ensure the convergence for finding a numerical solution, the following condition for the selected point should be checked $\sigma_{n}<\sigma_{A}\left(\varepsilon_{n}\right)$, according to the asymptotic limit in Equation (51) for the hardening stage.

**Figure 4:**
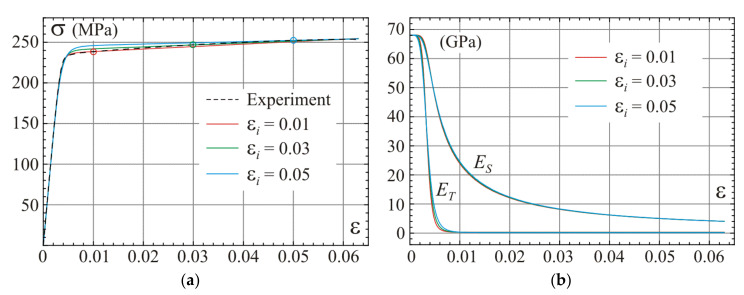
Influence of selecting the calibration point on the hardening stage for the one-dimensional model. (**a**) Calibrated constitutive relations, (**b**) secant and tangent moduli.

Graphs of the one-dimensional constitutive relation given by Equation (36) for three calibration points included in Table 1 are shown in Figure 4a. Distributions of the secant and tangent moduli from Equation (40) are presented in Figure 4b. We can observe graphically some sensitivity of stress–strain curves to the assumed location of the calibration point for determination of the hardening modulus E. The best agreement between the one-dimensional model prediction and the experiment is for a calibration point located between strain 0.01 and 0.02 for the regarded alloy.

**Figure 5:**
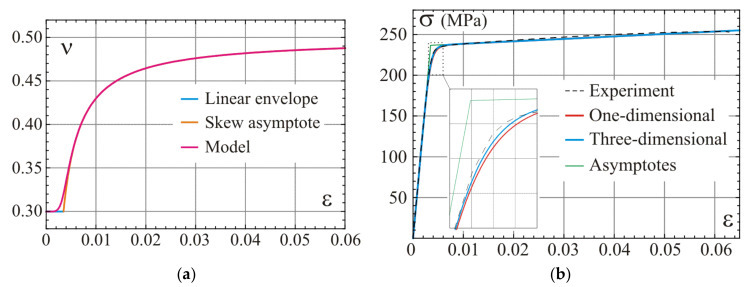
(**a**) Poisson’s ratio for the model and its envelope, (**b**) stress–strain relation for the one-dimensional and the uniaxial tension of the three-dimensional model with asymptotes.

Results based on the values of material parameters according to Equation (59) are presented in Figure 5. Graphs of the model Poisson’s ratio as a function of strain governed by Equation (56) is shown in Figure 5a. The lower band Poisson’s ratio for the linear envelope (asymptote at origin) according to Equation (47) and the function from Equation (55) for a skew asymptote of the uniaxial test curve are shown for comparison. Graphs in Figure 5b present comparison of the stress–strain curves determined for the one-dimensional model in Equation (36) with the uniaxial stress test of the three-dimensional model from Equation (56). Asymptotes are also included in the graphical interpretation of results. The model predictions (blue line) are in very good agreement with the experimental test (dashed line) used for the calibration. Besides the scatter of the experimental results close to the origin (for σ<40 MPa), the maximum relative difference between the model prediction and the experiment is up to 2% ($\left(\varepsilon_{Pred}-\varepsilon_{Exp}\right)/\varepsilon_{Exp}\leq 0.02$ when 40 MPa<σ<200 MPa), for the first stage, and 1% ($\left(\sigma_{Pred}-\sigma_{Exp}\right)/\sigma_{Exp}\leq 0.01$ when ε>0.002), for the second stage. 

### 3.4. Calibration Procedure for Fully Compressible Material

Procedure described in Section 3.3 will be now extended to the fully compressible material including the second stage of behavior, where the bulk modulus undergoes changes $K<K_{0}$. Results for the initial linear behavior governed by Equation (47) remain true. The present calibration is based on uniaxial experimental data. For the asymptotic relation in Equation (21), we use here a generalized Poisson’s ratio $\bar{\nu }$ ($\varepsilon_{T}=-\bar{\nu }\;\varepsilon$), which allows to express Equations (43) and (44) in the form:

(60) $$\sigma_{A}=\left[K\left(1-2\bar{\nu }\right)+\frac{4}{3}G\left(1+\bar{\nu }\right)\right]\varepsilon +\frac{\varepsilon }{Q_{AUX}}\left[\left(K_{0}-K\right)\left(1-2\bar{\nu }\right)+\frac{4}{3}\left(G_{0}-G\right)\left(1+\bar{\nu }\right)\right],\;$$

(61) $$0=K\left(1-2\bar{\nu }\right)-\frac{2}{3}G\left(1+\bar{\nu }\right)+\frac{1}{Q_{AUX}}\left[\left(K_{0}-K\right)\left(1-2\bar{\nu }\right)-\frac{2}{3}\left(G_{0}-G\right)\left(1+\bar{\nu }\right)\right],$$

where:

(62) $$Q_{AUX}=\sqrt{\left[\frac{\left(K_{0}-K\right){\left(1-2\bar{\nu }\right)}^{2}}{2\left(G_{0}-G\right)}+\frac{2{\left(1+\bar{\nu }\right)}^{2}}{3}\right]\frac{\varepsilon^{2}}{q_{0}^{2}}}.$$

Equation (61) can be solved for $\varepsilon =\varepsilon_{A}$, separately for the uniaxial compression and tension. In case of tension, we obtain:

(63) $$\varepsilon_{A}=q_{0}\frac{3\left(K_{0}-K\right)\left(1-2\bar{\nu }\right)-2\left(G_{0}-G\right)\left(1+\bar{\nu }\right)}{2G\left(1+\bar{\nu }\right)-3K\left(1-2\bar{\nu }\right)}P\left(\bar{\nu }\right),$$

which, when substituted into Equation (60), results in:

(64) $$\sigma_{A}=q_{0}\frac{6\left(K_{0}G-G_{0}K\right)\left(1-2\bar{\nu }\right)\left(1+\bar{\nu }\right)}{2G\left(1+\bar{\nu }\right)-3K\left(1-2\bar{\nu }\right)}P\left(\bar{\nu }\right),$$

where:

(65) $$P\left(\bar{\nu }\right)=\sqrt{\frac{6\left(G_{0}-G\right)}{3\left(K_{0}-K\right){\left(1-2\bar{\nu }\right)}^{2}+4\left(G_{0}-G\right){\left(1+\bar{\nu }\right)}^{2}}}.$$

Functions of the asymptotic direct strain $\varepsilon_{A}\left(\bar{\nu }\right)$ and the asymptotic normal stress $\sigma_{A}\left(\bar{\nu }\right)$ define the stress–strain relation in the uniaxial test in the parametric form. The generalized Poisson’s ratio $\bar{\nu }$ can change within the limits $\nu_{0}\leq \bar{\nu }\leq \nu$.

**Figure 6:**
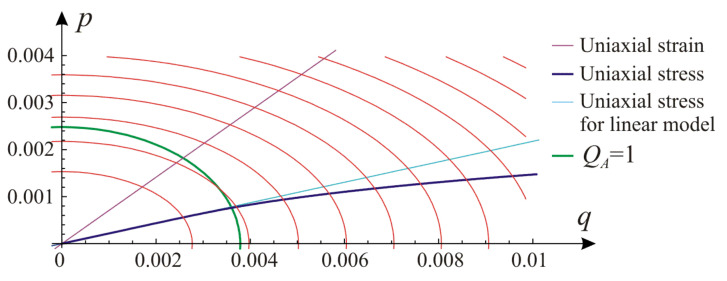
Contours of elastic strain energy for compressible nonlinear material for $K_{0}=56,700\;\mathrm{MPa}$, $G_{0}=26,200\;\mathrm{MPa}$, K=16,200 MPa, G=65 MPa, $q_{0}=0.00379$, n=3.44.

To find the calibration formulae in the following derivations, we will regard $\bar{\nu }$ as a parameter in the parametric description of the uniaxial stress state of the elastic model. The nonlinear path of the uniaxial stress in the plane of invariants $\left(p,q\right)$ is shown in Figure 6. Note that for a linear material, the uniaxial stress path goes along a straight line. For our model, the relation between $\varepsilon_{A}\left(\bar{\nu }\right)$ and $\sigma_{A}\left(\bar{\nu }\right)$ is nonlinear since the Poisson’s ratio function $\bar{\nu }$ changes with the loading level within the prescribed limits $\nu_{0}\leq \bar{\nu }\leq \nu$. This function has a skew asymptote which can be determined by calculation of the appropriate limits of the functions in Equation (63) and Equation (64) when $\bar{\nu }\to \nu$. The asymptote is described by the following function:

(66) $$\sigma_{AUX}=\frac{3\sqrt{2}\;q_{0}}{G+3K}\sqrt{\left(G_{0}-G\right)\left[\left(K_{0}-K\right)G^{2}+3\left(G_{0}-G\right)K^{2}\right]}+\frac{9GK}{G+3K}\varepsilon .$$

Comparison of the asymptote for the one-dimensional model from Equation (39) for tension with Equation (66) yields the following calibration formula:

(67) $$G=\frac{3EK}{9K-E}=\frac{E}{2\left(1+\nu \right)},$$

which confirms the definition of the hardening shear modulus (Equation (5)). Based on Equations (47) and (66), we can solve $\sigma_{L}\left(\varepsilon_{0}\right)=\sigma_{AUX}\left(\varepsilon_{0}\right)$ leading to the following dependence of $q_{0}$ on $\varepsilon_{0}$:

(68) $$q_{0}=\frac{3\left[\left(K_{0}-K\right)G_{0}G+3\left(G_{0}-G\right)K_{0}K\right]\varepsilon_{0}}{\left(G_{0}+3K_{0}\right)\sqrt{2\left(G_{0}-G\right)\left[\left(K_{0}-K\right)G^{2}+3\left(G_{0}-G\right)K^{2}\right]}}.$$

Equation (68) is the sought calibration formula for the fully compressible material.

The constitutive relationships for the uniaxial stress state described by Equations (43) and (44) can be expressed in a parametric way using the model Poisson’s ratio $\bar{\nu }$. In case of fully compressible material model, the relation between $\varepsilon \left(\bar{\nu }\right)$ and $\sigma \left(\bar{\nu }\right)$ is:

(69) $$\varepsilon \left(\bar{\nu }\right)=q_{0}P\left(\bar{\nu }\right)\;\sqrt[{2n}]{{\left[\frac{3\left(K_{0}-K\right)\left(1-2\bar{\nu }\right)-2\left(G_{0}-G\right)\left(1+\bar{\nu }\right)}{2G\left(1+\bar{\nu }\right)-3K\left(1-2\bar{\nu }\right)}\right]}^{2n}-1},$$

(70) $$\sigma \left(\bar{\nu }\right)=q_{0}\frac{6\left(G_{0}K-K_{0}G\right)\left(1+\bar{\nu }\right)\left(1-2\bar{\nu }\right)}{2\left(G_{0}-G\right)\left(1+\bar{\nu }\right)-3\left(K_{0}-K\right)\left(1-2\bar{\nu }\right)}\varepsilon \left(\bar{\nu }\right).$$

In the calibration procedure of the three-dimensional model of the fully compressible material, we assume the initial $\nu_{0}$ and the asymptotic ν Poisson’s ratios within the limits resulting from Equation (7). Note that $\nu_{0}$ and ν can be calibrated as well if experimental results for the transverse strain $\varepsilon_{T}$ are available. Next, we calculate the initial $E_{0}$ and the hardening E moduli from the uniaxial experimental data (direct strain versus stress). The initial modulus $E_{0}$ should be determined as a trend line at the initial part of an experimental stress–strain curve. The hardening modulus E is estimated from the pre-peak part (advanced hardening stage) of an experimental test. Then, we can calculate bulk and shear moduli $K_{0}$, $G_{0}$, K, G for both stages, and on the basis of the asymptotes of the one-dimensional model $\sigma_{0}$ or $\varepsilon_{0}$, we obtain $q_{0}$ from Equation (68). The last parameter n we determine from Equations (43) and (44) using one experimental point located in the vicinity of the elbow of a stress–strain curve.

For the regarded experimental data for aluminum AW6063 T66, the parameters $E_{0}$, E, $\sigma_{0}$ and $\varepsilon_{0}$ have the same values as in Section 3.3. Assuming values of the initial and the asymptotic Poisson’s ratios $\nu_{0}=0.3$ and ν=0.498 (almost incompressible asymptotic material behavior) located within the limits −0.994≤ν≤0.499, we calculate parameters of the three-dimensional model using Equation (5):

(71) $$K_{0}=56,700\;\mathrm{MPa},\;G_{0}=26,200\;\mathrm{MPa},\;K=25,200\;\mathrm{MPa},\;G=101\;\mathrm{MPa},$$

along with Equations (68) and (3):

(72) $$q_{0}=0.003691,\;p_{0}=\sqrt{\frac{2\left(G_{0}-G\right)}{3\left(K_{0}-K\right)}}\;q_{0}=0.002741.$$

The obtained values show significant drop in the bulk modulus value for the hardening stage if compared to asymptotic incompressible material model. Having results in Equations (71) and (72), we calculate the regularization parameter n using an additional calibration point, as previously, located in the vicinity of elbow of the experimental stress–strain curve $\left(\varepsilon_{n},\sigma_{n}\right)=\left(0.003475,214.5\;\mathrm{MPa}\right)$. Numerical solutions to the system of Equation (43) and Equation (44) are $\varepsilon_{T}=-0.001071$ and n=4.26≅4.

Again, the proposed calibration process is sensitive to the selection of calibration points. With the assumed ν=0.498, results of calibration for several locations of a point in the hardening zone of the experimental curve $\left(\varepsilon_{i},\sigma_{i}\right)$ are given in Table 2. The value of the regularization parameter n is also greatly affected by the choice of $\left(\varepsilon_{n},\sigma_{n}\right)$. The selected point should be below the asymptotic curve described parametrically by Equation (63) and Equation (64).

Contour lines of the elastic energy defined by Equation (1) in the plane of invariants $\left(p,q\right)$ for the fourth set of parameters (based on point with $\varepsilon_{n}=0.04$) from Table 2 are presented in Figure 6. A straight line representing the path of the uniaxial strain test and a curved path of the uniaxial stress according to Equation (42) with usage of Equation (69) and Equation (70) are shown as well. In case of a linear material, the uniaxial stress path follows a straight line (light blue), which is included for comparison. Because of the curved path of the uniaxial stress (dark blue), the calibration procedure is not straightforward, as was shown in this section. Location of the elbow ellipse $Q_{A}\left(p,q\right)=1$, separating the domain of the initial linear envelope from Equation (20) from the domain of the asymptotic relationship according to Equation (21), is also given with a solid green line in Figure 6.

**Figure 7:**
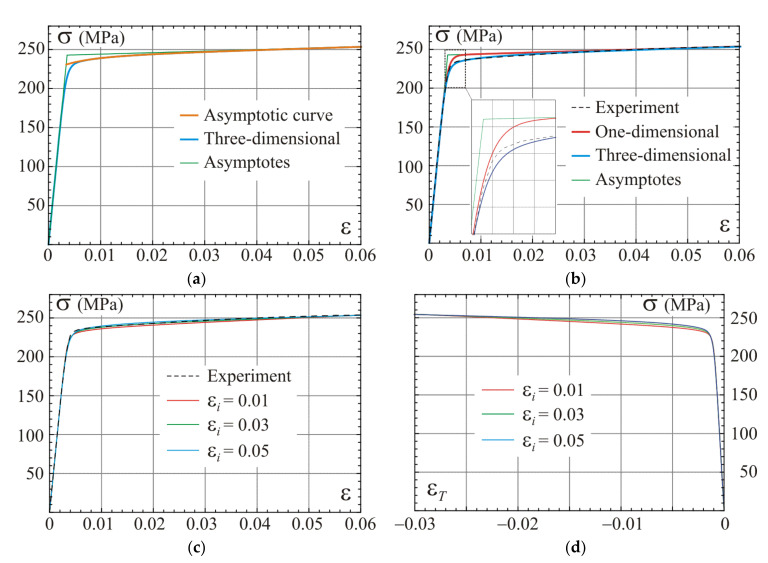
Stress–strain relation for compressible material: (**a**) asymptotic curve according to Equations (63) and (64) with asymptotes, (**b**) one-dimensional model and uniaxial tension of the three-dimensional model with asymptotes and experiment; and (**c**) comparison of calibrated three uniaxial tension curves with experiment, (**d**) comparison for transverse strain for three selected calibration points.

The stress–strain curves of the calibrated model for the fourth set ($\varepsilon_{n}=0.04$) of parameters from Table 2 are presented in Figure 7a,b. The calibrated constitutive relation for the uniaxial stress in the three-dimensional model according to the parametric formulae in Equations (69) and (70) is shown in Figure 7a. The asymptote of this relation described by Equation (66) and the asymptotic curve based on definitions in Equations (63) and (64) are plotted in this figure as well. Only in the vicinity of the elbow, the difference between curves is significant. The asymptotic curve (orange line) and its asymptote (green line) diverge in the elbow zone more than the curve of the one-dimensional model shown in Figure 7b. This effect is closely related to the nonlinear path of the uniaxial stress state shown in Figure 6. Generally, the bigger hardening in the second stage is, the larger difference between those curves can be observed. The stress–strain curves determined for the one-dimensional model in Equation (36) and for the uniaxial stress test of the three-dimensional model described by Equations (69) and (70) are compared to the experimental relation in Figure 7b. A very good compatibility between the experimental data and model predictions can be observed. The maximum error between the model’s prediction and the experiment is up to 2%, but generally decreases. Comparison of predictions according to the three sets of calibrated parameters included in Table 2 are presented in Figure 7c,d. In case of compressible material, stress–strain curves are less sensitive to the selection of the calibration point than those for asymptotically an incompressible material or one-dimensional model (Figure 4). Relations between the transverse strain and the stress are shown in Figure 7d. In contrast to calibration from Section 3.3, the best correspondence of the uniaxial test prediction and the experiment is for a calibration point located between strain 0.045 and 0.055 for the regarded alloy.

As an alternative to the presented equations, the calibration of the fully compressible material can be done by usage of four appropriately selected experimental points without reference to the one-dimensional model. In this approach, $\nu_{0}$ and ν must be assumed according to the limits set by Equation (7). Next, using the selected points from the experimental stress–strain curve, the four remaining parameters $E_{0}$, E, $q_{0}$ and n can be calculated. Based on Equations (43) and (44), a system composed of eight nonlinear equations, two for each calibration point, is solved numerically using Wolfram Mathematica. Four values of $\varepsilon_{T}$ (for each point) are determined as well. The solution is very sensitive to the choice of experimental points and starting points for iterations. Assuming Poisson’s ratios $\nu_{0}=0.3$, ν=0.498 and the experimental calibration points $\left(\varepsilon ,\sigma \right)$: $\left(0.00213,145\;\mathrm{MPa}\right)$, $\left(0.00378,223\;\mathrm{MPa}\right)$, $\left(0.00545,235\;\mathrm{MPa}\right)$, $\left(0.0606,254\;\mathrm{MPa}\right)$, the following values of parameters are found: $E_{0}=68,280\;\mathrm{MPa}$, E=153 MPa, $\sigma_{0}=245\;\mathrm{MPa}$ and n=3.94. Then, the parameters of the three-dimensional model can be obtained: $K_{0}=56,900\;\mathrm{MPa}$, $G_{0}=26,260\;\mathrm{MPa}$, K=12,750 MPa, G=51 MPa, $q_{0}=0.00382$, $p_{0}=0.00240$.

**Figure 8:**
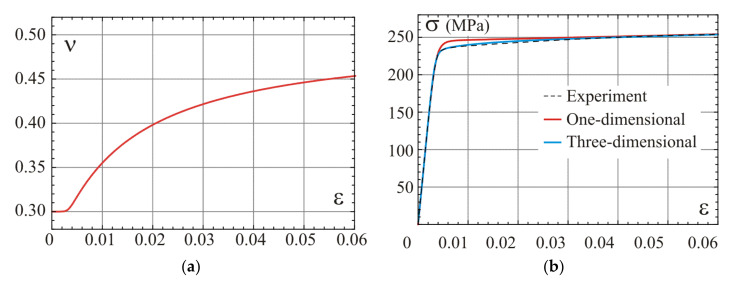
(**a**) Poisson’s ratio for the compressible model, (**b**) stress–strain relation for the one-dimensional model and the uniaxial tension of the three-dimensional model with asymptotes.

Results of this variant of calibration are shown in Figure 8. A graph of the model Poisson’s ratio as a function of strain (Equation (69)) is shown in Figure 8a. Comparing this graph with graphs presented in Figure 5a, we can observe a slower development of the model’s Poisson ratio for the fully compressible material than for the second stage of the asymptotically incompressible material. Figure 8b provides a comparison of the stress–strain curves determined for the one-dimensional model from Equation (36) and the uniaxial stress test of the three-dimensional model defined by Equations (69) and (70). The compatibility of the model’s predictions and the experimental curve is satisfactory. The difference between the one-dimensional model and the uniaxial stress for strains above the elbow is related to the nonlinear path of the uniaxial stress state shown in Figure 6. The best compatibility between the uniaxial test prediction and the experiment is when the third calibration point is selected between strain 0.045 and 0.055 for the regarded alloy. This range is in contradiction with the suggested range 0.01 and 0.02 for the asymptotically incompressible material.

## 4. Model Implementation in ABAQUS Environment with Basic Tests

In this section, we describe implementation of the proposed three-dimensional model and present a basic test of its correctness. The model of nonlinear elasticity introduced in Section 2 has been programmed in FORTRAN 90 (Intel Visual Fortran Compiler, Professional Edition 11.1) as a part of the user material procedure (UMAT) of ABAQUS/Standard [32]. The format of the UMAT procedure requires an incremental form of constitutive relationships, thus, we need to know an explicit definition of the fourth-order tensor of tangent stiffness. In the context of continuum mechanics, this relationship is compatible with the objective hypoelasticity relationship, which is formulated as the Zaremba–Jaumann objective derivative of the Kirchhoff stress tensor related to the rate of deformation tensor [21,25,26,27]. In case of small displacement theory, the rate of the Cauchy stress tensor is tied to the rate of infinitesimal strain tensor as it is given in Equation (25) and the tensor of tangent stiffness is described by Equation (27). In that case, the objectivity assumption is no longer valid. The tangent stiffness tensor is employed to obtain the structural tangent stiffness matrix which is further used for iterations according to the Newton–Raphson method. The constitutive relationship formulated by Equation (12) allows to determine the actual stress tensor at the end of an increment. More details on the finite element algorithm are described in [21,32,33]. The objectivity issues for FEM formulations are analyzed and discussed in [25,26,27]. In the ABAQUS/Standard, the moderately large deformation formulation is based on changing the infinitesimal stress and strain tensors to the Cauchy stress tensor and logarithm of the left deformation tensor (lnV). Such a relationship is not objective, so, for example, in a shear test, we obtain oscillations of the stress component, compare Figure 14.19 in [26]. It is also known that consideration of plastic properties of a material may lead to regularization of this response (with no oscillation).

We verify the developed UMAT procedure on boundary value problems with homogeneous fields of stress and strain. The uniaxial stress state, the uniaxial strain state and shear test are chosen as first tests. In both problems, we apply stretching in $x_{1}$ direction of cubic samples, modelled with one C3D8 finite element of ABAQUS. The selected problems can be solved analytically (uniaxial strain state) or partly analytically and partly numerically (uniaxial stress state) and compared with the results obtained using the considered model.

**Figure 9:**
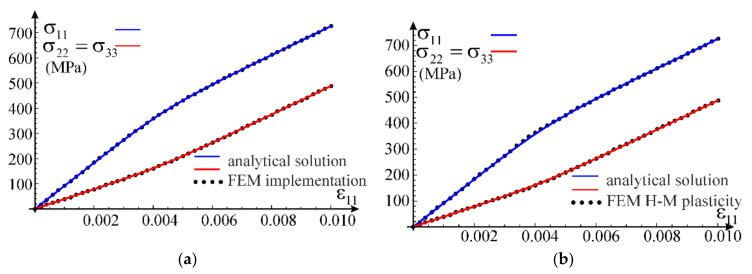
Comparison of the analytic elastic solution for the uniaxial strain test with: (**a**) FEM solution according to our own implementation, (**b**) FEM solution based on the standard elastic-plastic model.

In case of the uniaxial strain state, it is possible to determine analytic formulae for the components of stress tensor as a function of the direct strain component using constitutive relationship in Equation (12). This allows to compare the numerical solution obtained via the ABAQUS program with the exact one, see Figure 9a. Based on the calibration performed in Section 3.3 for the simplified model, the following values of material parameters have been adopted for the test: $G_{0}=26,200\;\mathrm{MPa}$, $K_{0}=K=56,700\;\mathrm{MPa}$, G=100 MPa, $q_{0}=0.003688$, $p_{0}=1.0$ and n=3. Additionally, we confront the received curves with predictions of the standard model of elastic-plastic material available in ABAQUS, see Figure 9b. This model is based on the Huber–Mises yield condition with isotropic hardening. The plastic hardening function is determined from a uniaxial tensile test. For this purpose, we assume $E_{0}=68,090\;\mathrm{MPa}$, $\nu_{0}=0.3$ and a piecewise linear plastic hardening function $\sigma \left(\varepsilon_{p}\right)$, where $\varepsilon_{p}$ is an equivalent plastic strain. This function is assessed from the own experiment of uniaxial tension of AW 6063 T66 aluminum alloy (Metpartner, Poland) to reproduce closely the observed stress–strain curve.

Comparison of both numerical simulations with the analytical solution is presented in Figure 9, for both axial and lateral stress components. It can be concluded that the nonlinear elasticity model has been correctly implemented; agreement of results is up to six digits. Additionally, comparing the exact solution obtained within the framework of nonlinear elasticity with the FEM solutions, it can be noticed that all obtained curves coincide; the results are fully compatible, qualitatively and quantitatively. In order to verify stability of solutions, numerical simulations are carried out for a wider range of deformations ($\varepsilon >\varepsilon_{U}=0.06064$) than the stable behavior of the tested aluminum alloy, which was used for the calibration.

**Figure 10:**
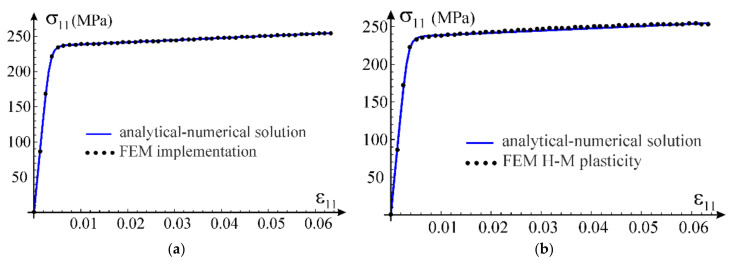
Comparison of the analytical-numerical nonlinear elastic solution for the uniaxial stress test with: (**a**) FEM solution according to own implementation, (**b**) FEM solution based on standard elastic-plastic model.

In case of the uniaxial stress test, it is not possible to obtain an explicit analytical closed-form solution for the considered material model. Only a parametric description of the relationship between axial stress and strain can be obtained, in which the model’s Poisson’s ratio is the parameter according to Equations (69) and (70). In order to verify the parametric description, the solution was obtained using the FindRoot nonlinear equation solving procedure by Wolfram Mathematica. From the condition of null transverse normal stress in Equation (44), a nonlinear relationship between the direct strain and the transverse strain was obtained. This allowed to determine the relationship between strain components, which in turn made it possible to obtain the normal stress in the stretching direction according to Equation (43). We will call this solution analytical-numerical and treat it as a reference for the FEM solution obtained using the proposed nonlinear elastic material model implemented in the UMAT procedure. Comparison of the two outcomes is presented in Figure 10a. Again, the compatibility of the results is excellent. The Huber–Mises model estimates are also qualitatively and quantitatively correct but the convergence is worse than that of our own model, see Figure 10b.

**Figure 11:**
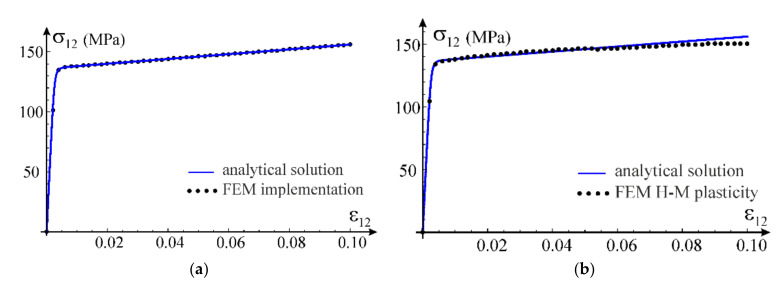
Comparison of the analytic nonlinear elastic solution for the pure distortion test with: (**a**) FEM solution according to our own implementation, (**b**) FEM solution based on standard elastic-plastic model.

The third test used for verification of the model implementation is the shear test. The calculations are carried out for one C3D8 finite element. Appropriate displacements are applied in the element’s nodes to induce a purely distortional deformation. In case of our own implementation, the obtained results are presented against the analytical solution in Figure 11a. Comparison of the exact solution for the nonlinear elastic model with results of the numerical test according to the Huber–Mises elastic-plastic model is shown in Figure 11b. In this case, the model prediction is compatible with the analytic one only for a limited range of the distortional strain. With an increasing strain level, the discrepancy between solutions increases. 

## 5. FEM Solutions to Selected Boundary Value Problems

In this section, we regard three boundary value problems which can be seen as structural element models. We analyze a rod under self-weight load, a rectangular plate with a circular hole in tension and a compressed column. In case of the rod, we compare an exact solution with the numerical one, which is based on the proposed model. Those examples serve as preliminary applications of the developed model to solutions of more realistic engineering structures with inhomogeneous fields of stress and strain.

### 5.1. Rod Stretched under Its Self-Weight

**Figure 12:**
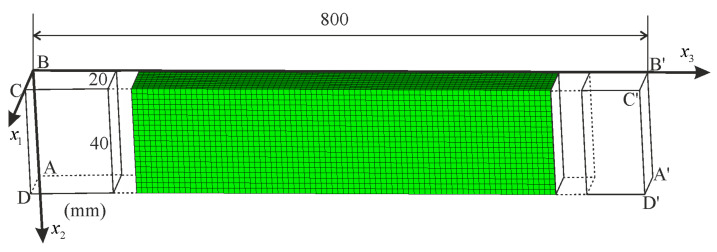
Geometry and FEM mesh of the regarded rod problem.

A problem of a rod subjected to tensile load (self-weight) is now regarded. The rod with dimensions 20 mm × 40 mm × 800 mm is meshed with 80,000 finite elements of C3D8 type (spatial, eight-node with linear shape functions). The resulting FEM mesh is uniform as shown in Figure 12. For nodes located on the ABCD surface, the displacement component in direction 3 is blocked, and for the node at point B, the displacement components in directions 1 and 2 are blocked as well. It is assumed that gravity acts in the direction of axis 3 and the self-weight load is realized by assigning volumetric forces applied to elements, using the scaled mass density of the rod. The prescribed load is assumed in such a way that the ultimate stress $\sigma_{33}=\sigma_{U}=253.9\;\;\mathrm{MPa}$ is reached in the most stressed cross-section, that is $x_{3}=0$. In this case, we consider a reduced model of nonlinear elasticity neglecting the hardening stage behavior. This assumption is undertaken to compare numerical simulation with an existing analytical solution to the problem. Consequently, the following material data are adopted for the numerical calculations: $G_{0}=26,200\;\mathrm{MPa}$, $K_{0}=56,700\;\mathrm{MPa}$, G=K=0, $q_{0}=0.004252$, $p_{0}=0.002358$ and n=2.58. Those values of material parameters were obtained to fit the one-dimensional model to the initial stiffness and the ultimate stress of the regarded experimental test. 

The solution of the rod in tension under self-weight (or in the case of a one-dimensional model under the action of a uniformly distributed load) is of fundamental importance, because it is one of few problems with inhomogeneous displacement, stress and strain fields for which an analytical solution can be found. For this purpose, hardening in the second phase of deformation must be excluded from the proposed model. Thus, neglecting hardening (E=0) in the one-dimensional model proposed in Section 3.1, the constitutive relationship defined in Equation (36) can be inverted. Then, the resulting formula for the strain function can be integrated to find the displacement function. The solution to this problem is described by the following functions:

(73) $$\sigma_{33}\left(x_{3}\right)=\frac{\mu }{A}\left(L-x_{3}\right),\;\varepsilon_{33}\left(x_{3}\right)=\frac{\mu N_{U}\left(L-x_{3}\right)}{E_{0}A\;\sqrt[{2n}]{N_{U}^{2n}-\mu^{2n}{\left(L-x_{3}\right)}^{2n}}},$$

(74) $$u_{3}\left(x_{3}\right)=\frac{\mu L^{2}}{2E_{0}A}F\left[\frac{1}{2n},\frac{1}{n},1+\frac{1}{n},{\left(\frac{\mu L}{N_{0}}\right)}^{2n}\right]-{\left(1-\frac{x_{3}}{L}\right)}^{2}F\left[\frac{1}{2n},\frac{1}{n},1+\frac{1}{n},{\left(\frac{\mu \left(L-x_{3}\right)}{N_{0}}\right)}^{2n}\right]$$

for $0\leq x_{3}\leq L$, compare Figure 12. In the above functions, μ=ρAg is the load uniformly distributed along the rod’s length, ρ is the mass density, g is the gravitational acceleration, A is the cross-section area, L is the rod’s length, and F is the hypergeometric function. $N_{U}=\sigma_{U}A$ is the load capacity of the rod’s cross-section, while $\mu_{U}=N_{U}/L$ is the limiting load of the rod. Note that by setting n=1, we can obtain the simplest solution to the problem:

(75) $$\varepsilon_{33}\left(x_{3}\right)=\frac{\mu N_{U}\left(L-x_{3}\right)}{E_{0}A\;\sqrt{N_{U}^{2}-\mu^{2}{\left(L-x_{3}\right)}^{2}}},\;u_{3}\left(x_{3}\right)=\frac{N_{U}}{E_{0}A\mu }\left[\sqrt{N_{U}^{2}-\mu^{2}{\left(L-x_{3}\right)}^{2}}-\sqrt{N_{U}^{2}-\mu^{2}L^{2}}\right].$$

Using the limiting load $\mu_{U}$, it takes the shortest form:

(76) $$\varepsilon_{33U}\left(x_{3}\right)=\frac{N_{U}\left(L-x_{3}\right)}{E_{0}A\;\sqrt{x_{3}\left(2L-x_{3}\right)}},\;u_{3U}\left(x_{3}\right)=\frac{N_{U}}{E_{0}A}\sqrt{x_{3}\left(2L-x_{3}\right)}.$$

**Figure 13:**
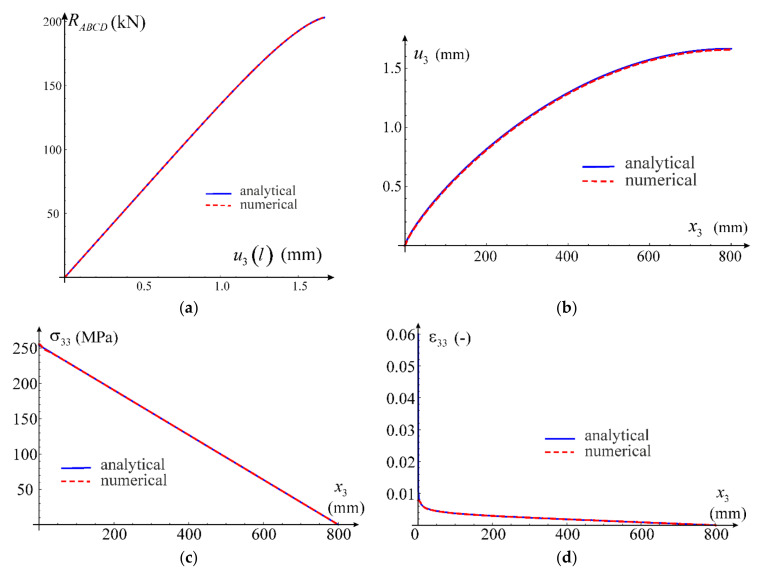
Comparison of analytical and numerical solutions for the rod: (**a**) reaction versus maximum displacement, (**b**) displacement, (**c**) stress, (**d**) strain.

Comparison of the results obtained using various methods is presented in Figure 13. Relation between the reaction force $R_{ABCD}$ of the fixed end and the maximum displacement of the rod’s free end is given in Figure 13a. It shows an excellent correlation with the analytic solution. In case of displacement (Figure 13b), the FEM solution provides slightly lower values. For the $\sigma_{33}$ stress component, great compatibility of the results can be observed in the entire rod’s domain, as shown in Figure 13c. The functions describing the strain component $\varepsilon_{33}$ (Figure 13d) are in good agreement besides the zone near $x_{3}=0$. At the fixed end of the rod, the analytical solution for strain changes significantly when the load approaches the ultimate value $\mu_{U}$. Since we have assumed a uniform mesh with a side length of an element equal to 2 mm, it is not possible to obtain such a rapid change in distribution of strain. Certainly, a refinement of the mesh in this zone would significantly improve the FEM model predictions, but our goal here is to illustrate a general correctness of the implementation in case of a complex problem with inhomogeneous displacement, strain and stress fields, so we leave further improvements out.

**Figure 14:**
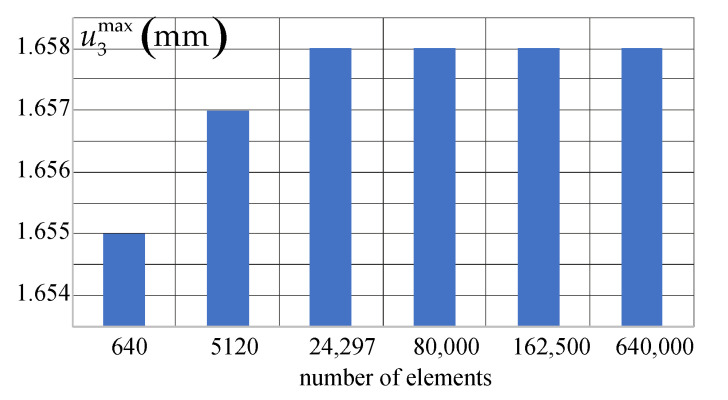
Mesh sensitivity in the FEM solution of the rod problem.

We have performed a convergence analysis of the numerical solution using six meshes as depicted in Figure 14. The bar chart presents the maximum displacement $u_{3}$ (at the tip of the rod) as a function of the number of elements. The obtained results show an excellent convergence rate for the regarded structure. This test confirms very good stability and accuracy of the computational model even for a coarse mesh. In contrast to the elastic-plastic models, the nonlinear elastic models are less sensitive to the localization of deformation. This feature depending on the problem analyzed can be regarded as an advantage or a disadvantage.

### 5.2. Rectangular Plate with a Circular Hole

**Figure 15:**
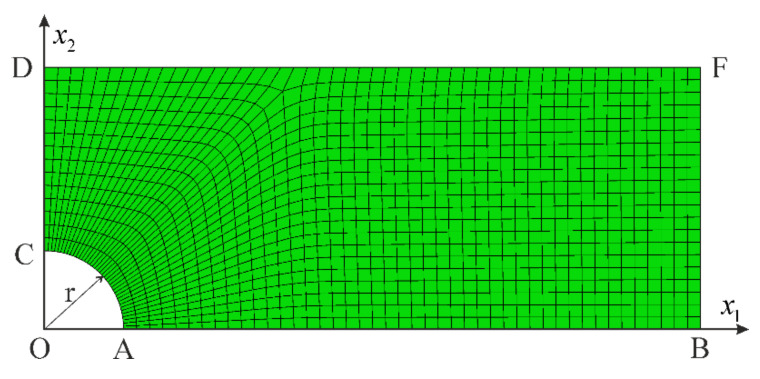
FEM mesh and characteristic points of the rectangular plate with a circular hole.

The next test problem is a rectangular plate with a circular hole in its center. The plate is stretched along the $x_{1}$ direction according to Figure 15. The existence of the hole results in inhomogeneous fields of displacement, strain and stress and emerging stress concentration effects. Considering symmetry of the problem, we can analyze the computational model reduced to a quarter as it is shown in Figure 15. The modeled region has dimensions 50 mm × 20 mm (DF × BF) with the hole of radius 6 mm and the plate thickness of 2 mm.

**Figure 16:**
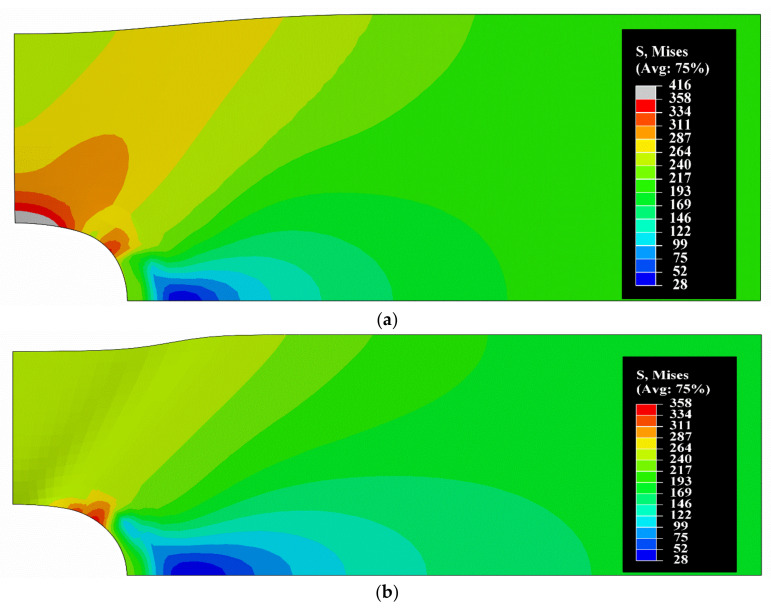
Contour plots of Huber–Mises equivalent stress (MPa) under the applied displacement $u_{1}^{BF}=2\;\mathrm{mm}$ in the considered plate for: (**a**) introduced nonlinear elastic model, (**b**) Abaqus elastic-plastic model.

In order to preserve the symmetry of the problem, the boundary conditions $u_{1}=0$ along the CD edge and $u_{2}=0$ on the AB edge are assumed. The stretch of the plate is driven by enforcing the displacement $u_{1}=2\;\mathrm{mm}$ on the BF line. The FEM mesh of the plate, shown in Figure 15, consists of 3531 8-node spatial elements with 3 elements along the thickness. We assume that the plate is made of the aluminum alloy considered in Section 4 and adopt the same values of material parameters. The problem is solved according to the proposed nonlinear elasticity constitutive model and using the standard elastic-plastic model of ABAQUS. We regard two cases of the problem settings, one within the framework of small deformations, and the other one of moderately large deformations, with usage of the NLGEOM option, compare in [21,25,32]. Contour plots of the Huber–Mises equivalent stress for those two cases are presented in Figure 16. Solution according to the proposed nonlinear elastic model is shown in Figure 16a, while results obtained using the elastic-plastic model are given in Figure 16b. The maximum stress on the stress scale is associated to the solution with usage of the standard elastic-plastic model. Therefore, for the nonlinear elastic model solution, the gray sub-area on the stress scale represents stresses greater than the maximum stress obtained in the elastic-plastic solution. We can notice a difference in the shape of the deformed plate between the obtained solutions. Due to possible stress redistribution in the elastic-plastic model, there is a significant change in the zone of stress concentration if compared to the nonlinear elastic model.

**Figure 17:**
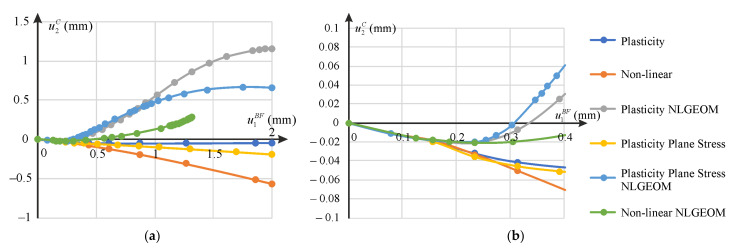
Displacement $u_{2}$ of node C as a function of the edge displacement BF: (**a**) $u_{1}^{BF}\in \left[0,2\right]\left(\mathrm{mm}\right)$, (**b**) $u_{1}^{BF}\in \left[0,0.4\right]\left(\mathrm{mm}\right)$.

**Figure 18:**
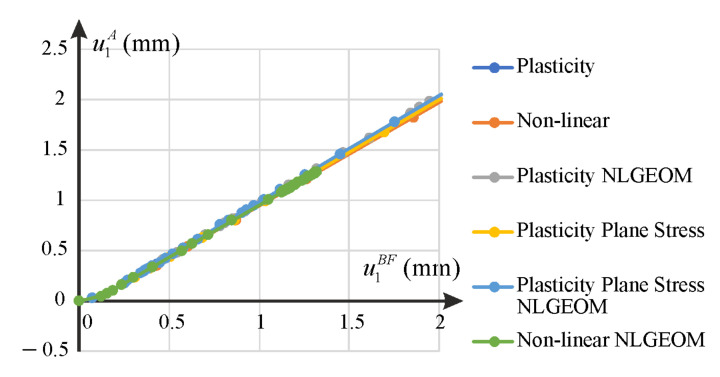
Displacement $u_{1}$ of node A as a function of the edge displacement *BF*.

Graphs of the displacement component $u_{2}$ of the node C as a function of the imposed displacement of the BF edge are shown in Figure 17. We applied a quite large deformation $u_{1}^{BF}$ to investigate possible differences in predictions for various models and settings. We can observe that depending on the constitutive model used for simulations, the presented graphs may change significantly. Solutions according to the nonlinear elastic, the elastic-plastic and the elastic-plastic plane stress models in the small strain setting are described by monotonically decreasing functions. In other cases, we observe a more complex dependence between the regarded displacements. In case of nonlinear elastic model, with the NLGEOM option turned on, the response rapidly stiffens and it was not possible to find a solution to the problem in the entire range of the assumed boundary displacement. This locking effect is caused by the increasing Poisson’s ratio (close to $\nu_{\max}$ limit set in Equation (7)) with extensive straining of the plate. Graphs of the displacement component $u_{1}$ of the node A as a function of the displacement prescribed on the BF edge are presented in Figure 18. Dependencies between the functions are almost identical for all the considered constitutive models and formulations. Based on these results, it can be concluded that the responses obtained using the proposed nonlinear elastic model in the framework of small strain theory are stable and monotonic over the entire range. The dominant effects for the investigated $u_{1}$ displacement of node A coincide with the results achieved by the elastic-plastic models. However, it should be emphasized that the automatic generalization of the nonlinear elastic model to moderately large deformations via the NLGEOM option does not lead to a stable behavior. Using material data as for small deformation theory, we obtained the effect of rapid stiffening of the response, which leads to the lack of convergence of the standard incremental algorithm of ABAQUS. Markers placed along the curves represent solutions in the successive increments; their increasing density informs about convergence problems, and, inversely, increasing distance between them indicates improved rate of convergence. However, when comparing the solutions obtained according to the elastic-plastic model for small and moderately large deformations, it can be noticed that turning on the NLGEOM leads to rapid changes, not only in values, but also in character of the response function (Figure 17). As it can be seen in Figure 17b, for very small deformation, the $u_{2}^{C}$ prediction is the same for all analyzed models. For substantial deformation, one can observe the displacement oscillation symptoms. This type of behavior is typical for small deformation theory used for large deformation (or/and rotations) [25,26]. The fact that in the case of the proposed nonlinear elastic model the $u_{2}^{C}$ function is monotonic in the whole range of deformation confirms its model. Of course, at this stage of research, it is too early to formulate any solid conclusions, but we plan to deal with the problem of model’s application for large deformation theories in the future.

**Figure 19:**
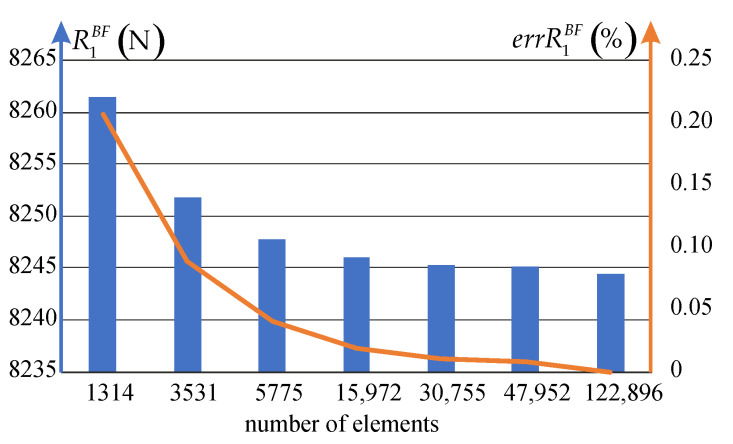
Mesh sensitivity of the FEM solution for the plate problem.

Mesh sensitivity for the analyzed plate is investigated as well. In contrast to the rod under self-weight in case of the plate, we compare values of the total reaction since the loading is applied through the displacement enforcement. The value of this reaction for the increasing number of elements is shown in Figure 19. We can see that better results may be obtained for meshes with larger number of elements, but when calculating the relative error (for which the solution obtained for 122,896 elements is treated as the reference), one can observe that even for the coarse mesh, consisting of 3531 elements, the error is less than 0.2% when compared to the total reaction obtained for the fine mesh consisting of 122,896 elements.

### 5.3. Compression of Column with Initial Shape Imperfection

The last analyzed problem is a column subjected to compression. Geometry of the column is the same as regarded in Section 5.1 (Figure 12). Material data are the same as in Section 4. Boundary conditions on the *ABCD* and *A*′*B*′*C*′*D*′ planes are changed as follows. On the surface of *ABCD*, all the displacement degrees of freedom are blocked. On the *A*′*B*′*C*′*D*′ surface, the displacement components in direction 1 and 2 are blocked and the axial displacement of value $u_{3}=-80\;\mathrm{mm}$ in direction 3 is applied. Such boundary conditions represent a column fixed on both ends. The assumed axial loading may lead to buckling. That is why all below solved problems are formulated in the theory of moderately large deformations (NLGEOM option is active). We also introduce geometric imperfections of the column to effectively solve the regarded stability problem.

**Figure 20:**
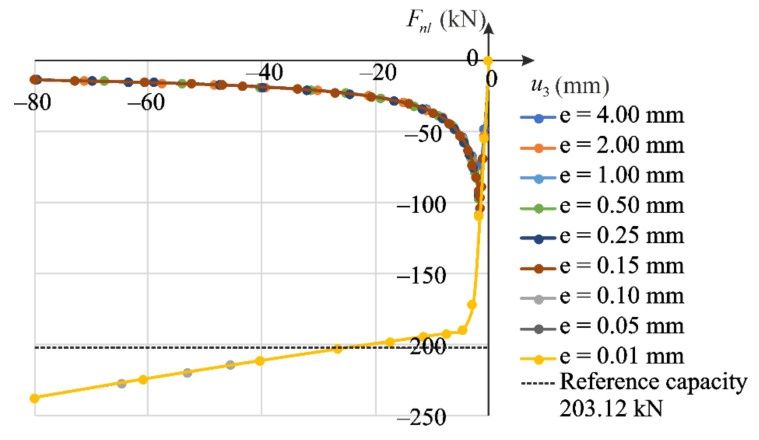
Axial force versus applied displacement for the nonlinear elastic model.

**Figure 21:**
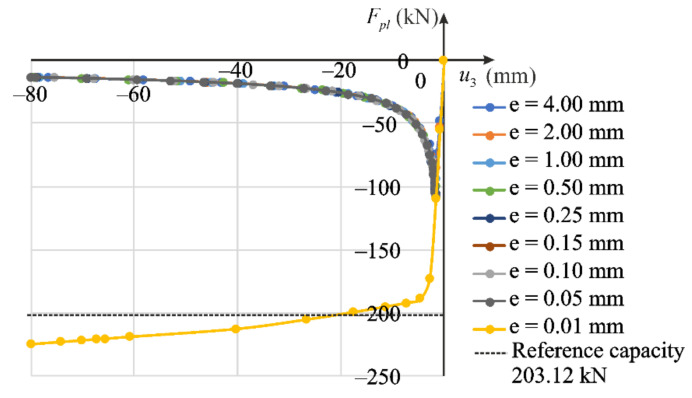
Axial force versus applied displacement for the elastic-plastic model.

Prior to the analysis of the column compression, the eigenvalue buckling problem was solved, in which ten buckling eigenmodes were determined (BUCKLE option in the ABAQUS/Standard). The first form of buckling governs the global column stability about the axis of minor inertia moment of the cross-section, that is in the $x_{1}x_{3}$ plane. This normalized buckling form is treated as the given imperfection. Next, the amplitude of the selected buckling mode is assumed. We solve nine stability problems with different magnitudes of imperfections. The first eigenmode is scaled with the following amplitudes of imperfection: e=0.01,0.05,0.10,0.15,0.25,0.50,1.00,2.00,4.00 mm. The equilibrium paths obtained for the individual imperfection values according the proposed nonlinear elastic model are shown in Figure 20, while equilibrium paths for the elastic-plastic model are presented in Figure 21. Stable response of the column is observed for small imperfection amplitudes, while for larger imperfections buckling occurs. Axial forces $F_{nl}$ (for nonlinear elastic model) or $F_{pl}$ (for elastic plastic model) are calculated as total reactions exerting from the *A*′*B*′*C*′*D*′ surface due to the displacement applied to the boundary nodes. Their extreme values are associated with the critical force F. The reference capacity $F_{R}=A\sigma_{U}=203\;\mathrm{kN}$ representing the ultimate load determined for the experimental strength $\sigma_{U}=253.9\;\mathrm{MPa}$ of the regarded alloy is shown for comparison. 

**Figure 22:**
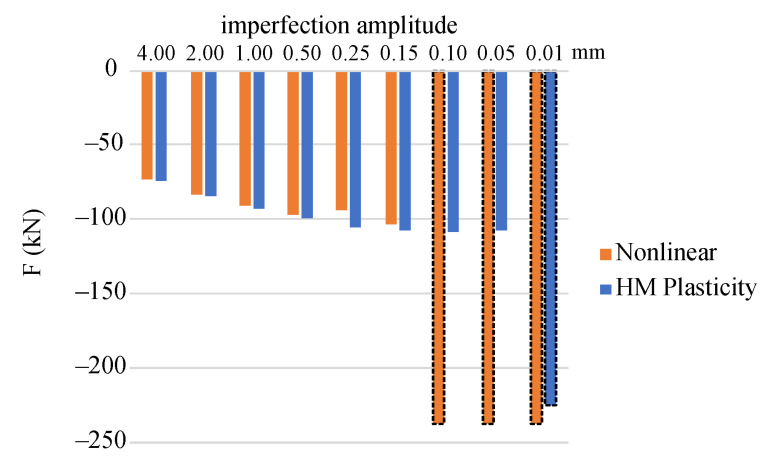
Critical force as a function of the initial amplitude of shape imperfection for the regarded constitutive models.

The bar graphs shown in Figure 22 represent values of the critical forces obtained using both constitutive models. In case of bars with a contour marked as a dashed line, no buckling occurred; for those columns a stable compression with bending is observed. We can notice that for the nonlinear elastic solutions, values of the obtained critical forces are slightly lower than the respective forces received for the considered elastic-plastic model. This fact is often discussed in the literature, where attention is paid to better compliance of solutions obtained within the framework of the theory of nonlinear elasticity (the deformation theory of plasticity) with experiments. Values of the critical forces according to the plastic flow theory are generally higher than the experimental ones [6,7,8]. According to the nonlinear elastic model, the columns have not buckled for the initial amplitude of shape imperfections 0.10, 0.05 and 0.01 mm. For the elastic-plastic model, this occurred only for the smallest amplitude value. This effect was reported in [8] as well. The discrepancy is due to almost linear hardening included in the nonlinear elastic model, which allows for a more stable column response when the imperfection is small enough.

**Figure 23:**
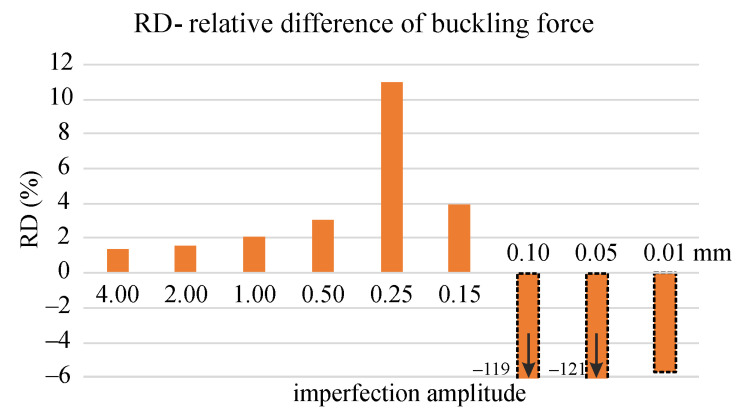
Relative difference between the critical forces obtained according to the nonlinear elastic and elastic-plastic model.

In order to effectively compare the obtained values of the critical forces, the relative difference defined as $RD=100\left(\min F_{pl}-\min F_{nl}\right)/\min F_{pl}$ is shown in Figure 23. In those cases where buckling took place, the differences did not exceed 4% for both constitutive models. An exception is the relative error exceeding 10% for 0.25 mm imperfection amplitude. It should be emphasized that in all analyzed cases, the standard algorithm of step division into increments implemented in the ABAQUS/Standard program was used. This means that its settings could influence both the values of the critical forces and the solution convergence. In order to exclude this issue from the analysis, the settings were always the same for both models.

**Figure 24:**
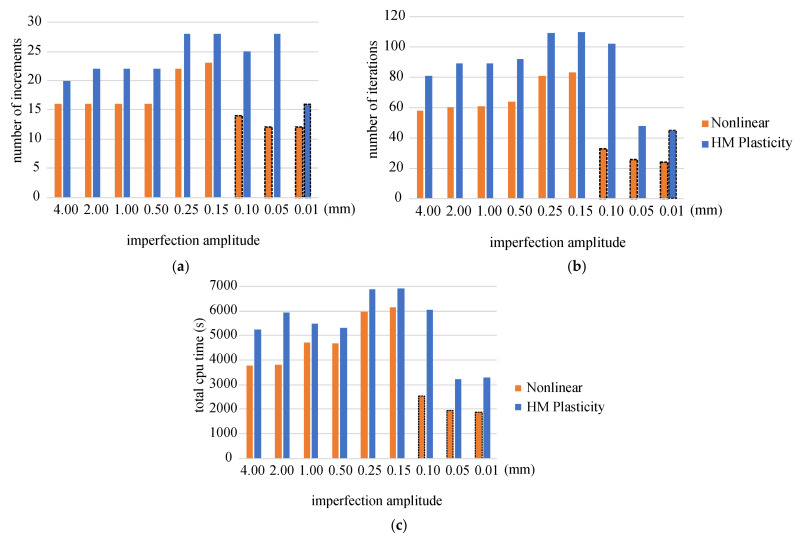
Number of: (**a**) increments, (**b**) iterations needed to solve the problem, (**c**) CPU time needed to solve the problem as a function of the amplitude for geometric imperfection.

Additionally, the bar chart in Figure 24 presents information on the number of increments, iterations and CPU time needed to solve the regarded stability problems. The number of increments or iterations indirectly shows the rate of convergence in the calculation process. It can be seen that, in all cases, the solution for the proposed model is reached with a significantly smaller number of increments (iterations), despite the fact that in order to solve the problem, we use the standard FEM algorithm ABAQUS/Standard, which primarily supports the elastic-plastic constitutive model. The reduction in computational time is perhaps not spectacular, but in all cases, the time was shorter for our model than for the elastic-plastic one.

## 6. Summary

In the paper, a fully three-dimensional model of a nonlinear elastic material is formulated in the framework of Green’s elasticity, so restricted to the path-independent material behavior. In the obtained constitutive relationship, the Cauchy stress tensor is a function of the strain tensor. The relation is non-invertible, what entails some difficulties in the calibration process when stress tests are used, but is excellent for strain-driven tests and convenient to finite element implementation. The model’s predictions are governed by two-stage material behavior. During the first stage (low strain level) the stress–strain relation is close to the linear Hooke’s law. After a smooth transition, in the second stage (high strain level), an almost linear hardening occurs. This type of response can be applied to many metallic materials, although here we have regarded only a low-hardening aluminum alloy. In contrast to the standard plastic incompressibility in elastic-plastic models, the proposed model can describe material compressibility in both stages of material behavior. As a result, the variability of Young’s modulus and Poisson’s ratio with strain or stress are inseparably included in the model formulation. The model requires six material constants: two bulk moduli $K_{0}$ and K; two shear moduli: $G_{0}$ and G; the elbow strain $q_{0}$ marking the stage switch, and the regularization parameter n. Material constant n is responsible for the smooth transition from the first stage to the second stage of material response.

The presented model is reasonably flexible, in the second stage, it can be reduced to obtain the asymptotically incompressible response. Since the derived constitutive relationship is in the format $\sigma \left(\varepsilon \right)$, it is convenient for finite-element method implementation, but it is less suitable for calibration of material parameters using stress tests. Those features are in contrast to models in the format $\varepsilon \left(\sigma \right)$, where a calibration based on stress tests is convenient, but finite element programming is problematic, compare in [21]. More complex constitutive relations, that reasonably reflect the real material behavior, are usually non-invertible. Therefore, the calibration process of the proposed model is discussed in details. We use the uniaxial test, which is the most often performed for aluminum, and employ a one-dimensional analog of the three-dimensional model. The various approaches shown for the calibration allow to effectively determine the material constants present in the model’s equations.

The finite element implementation of the proposed nonlinear elastic model has been performed in the ABAQUS environment via a UMAT subroutine. The developed code has been tested on several problems confirming its correctness and applicability in numerical simulations. After verification, we have performed a preliminary analysis of a global buckling problem. The advantages of the proposed model have been shown. As pointed out in [5], the material compressibility influences the values of the critical loads. Thus, taking into account compressibility in both elastic and elastic-plastic stages of the material response, which is not done for most classic models, the standard elastic-plastic model of ABAQUS included, may deliver new, improved estimates of the critical loads. Our future work is to focus on the application of the introduced model to engineering structures susceptible to buckling.

Application of the developed and coded model is not only restricted to buckling problems mentioned in this paper. It may be used to solve any problem which can be regarded as nonlinear elastic in the framework of infinitesimal strain theory of isotropic materials. The model predicts path-independent responses; thus, the stress redistribution effect or cyclic loading cannot be described properly. It is rather suitable for monotonic loading regimes, which are usually used in structural design procedures. The model can be successfully used in micro-level mechanics of materials to simulate grain (or micro-element) elastic response or employed in homogenization theory for determination of macroscopic generally anisotropic material properties.

## Figures and Tables

**Table 1:** Dependence of material parameters on selection of calibration point $\left(\varepsilon_{i},\sigma_{i}\right)$.

$\varepsilon_{i}$	$\sigma_{i}$	E	$\sigma_{0}$	$\varepsilon_{0}$	ν	G	$q_{0}$	n
%	MPa	MPa	MPa	%	-	MPa	%	-
1	238.6	302.3	235.6	0.3475	0.4991	100.8	0.3689	3.283
2	242.9	270.9	237.5	0.3502	0.4992	90.34	0.3717	3.049
3	246.7	235.1	239.6	0.3532	0.4993	78.42	0.3749	2.823
4	249.9	194.4	242.1	0.3566	0.4994	64.81	0.3785	2.605
5	252.3	150.4	244.8	0.3603	0.4996	50.14	0.3824	2.408

**Table 2:** Dependence of material parameters on selection of calibration point $\left(\varepsilon_{i},\sigma_{i}\right)$ in case of compressible material.

$\varepsilon_{i}$	$\sigma_{i}$	E	$\sigma_{0}$	$\varepsilon_{0}$	K	G	$q_{0}$	n
%	MPa	MPa	MPa	%	MPa	MPa	%	-
1	238.6	302.3	235.6	0.3475	25,190	100.9	0.3691	4.265
2	242.9	270.9	237.5	0.3502	22,570	90.41	0.3720	3.979
3	246.7	235.1	239.6	0.3532	19,590	78.48	0.3752	3.706
4	249.9	194.4	242.1	0.3566	16,200	64.87	0.3789	3.445
5	252.3	150.4	244.8	0.3603	12,530	50.19	0.3828	3.211

## Data Availability

Data sharing is not applicable to this article.
